# Coding for channels with intermittent feedback and its security analysis

**DOI:** 10.1371/journal.pone.0347790

**Published:** 2026-04-29

**Authors:** Jianwen Yang, Qiang Huang, Yating Lin

**Affiliations:** 1 Geely University of China, Chengdu, China; 2 School of Information Science and Technology, Southwest Jiaotong University, Chengdu, China; Beijing Institute of Technology, CHINA

## Abstract

The Schalkwijk-Kailath (SK) scheme for the AWGN channel with noise-free feedback is well-known since its coding complexity grows linearly with the coding blocklength, and it is capacity-achieving and has extremely lower decoding error probability comparing with existing excellent codes such as LDPC and Polar codes. However, extension of SK scheme to more practical scenarios is challenging since it depends heavily on the feedback success. This paper aims to investigate how to design SK-type schemes for channels with intermittent feedback and establish the relationship between the coding blocklength, the desired decoding error probability, the achievable transmission rate and secrecy level of our proposed schemes. Specifically, first, for the additive white Gaussian noise (AWGN) channel with intermittent noise-free feedback, a variation of the well-known Schalkwijk-Kailath (SK) scheme for the AWGN channel with noise-free feedback is proposed, and the corresponding achievable rate is characterized for given coding blocklength and decoding error probability. Subsequently, the proposed scheme is extended to channels with noisy intermittent feedback. To quantitatively evaluate the secrecy performance, we adopt the eavesdropper’s normalized equivocation as the secrecy metric and analytically characterize the achievable secrecy level of the proposed schemes. In particular, we show that perfect weak secrecy, i.e., asymptotically vanishing information leakage rate, can be achieved under certain conditions. Numerical results show that for a given decoding error probability threshold, our proposed schemes require lower signal-to-noise ratio and significantly shorter coding blocklength comparing with LDPC code. The study of this paper may provide a way to construct efficient coding scheme for channels in the presence of intermittent feedback.

## 1 Introduction

Ultra-reliable and low-latency communications (URLLC) is becoming one of the critical services in 5G and future 6G wireless communications [1–3] since it aims to guarantee high reliability levels, and requires coding scheme with significantly short coding blocklength due to strict latency constraint in several practical scenarios, such as road safety information and autonomous driving.

One possible effective solution to URLLC is feedback control based coding scheme, which was first proposed in [4], known as the Schalkwijk-Kailath (SK) scheme. In [4], the additive white Gaussian noise (AWGN) channel with noise-free feedback was studied, where the channel feedback helps the transmitter to construct a highly efficient coding scheme. In this scheme, at the first time instant, the message is directly transmitted over the AWGN channel, and the receiver adopts a zero-forcing method to do his first estimation about the message. By noise free channel feedback, the transmitter knows the message’s first estimation by the receiver, and sends the estimation error (difference between the estimation and the real message) at the second time instant. Once receiving the signal, the receiver applies linear minimum mean square estimation (LMMSE) to the received signal and obtains a new estimation about the estimation error at the last time instant, and then he updates his estimation about the message by using this new estimation and the initial estimation. By iteration, it was shown that the receiver’s estimation error about the message vanishes with the increasing of the coding blocklength. Later, [5] showed that the SK scheme is in fact a feedback control based scheme, and re-presented this scheme from control-theoretic aspect. Note that at each time instant, the encoding-decoding procedure of the SK scheme consists of 5 linear operations, which indicates that the coding complexity of the SK scheme grows linearly with the coding blocklength *N*, and can be approximately denoted as 𝒪(N). Besides this, for transmitting a bitstream with *K* bits, the SK scheme only requires at least 2 × *K* bits memory at both the transmitter and receiver. These indicate that the SK scheme has extremely low encoding-decoding complexity, which shows potential for channel coding in practical communication systems [6–10]. Besides this, in [4], it was shown that as the coding blocklength approaches infinity, the decoding error probability of the SK scheme declines doubly exponentially to zero, which indicates that to achieve a fixed decoding error probability, the coding blocklength of the SK scheme is significantly shorter than those of the well-known linear block codes, such as low-density parity-check (LDPC) codes and Polar codes.

Another interesting property of the SK scheme is that it satisfies the physical layer security (PLS) requirement by itself. Here recall that the PLS was first investigated by Shannon [11], and subsequently, Wyner [12] studied how to transmit a message over a noisy channel with perfect secrecy guaranteed. The secrecy capacity, which is the maximum transmission rate with perfect secrecy, was characterized. [13] showed that for the AWGN channel with noise-free feedback and an external eavesdropper, the SK scheme is the optimal secure scheme for such a model, which indicates that the SK scheme not only achieves the optimality, but also is self-secure. This self-secure property of the SK scheme has been extensively studied in literature, see [14–18].

Though the SK scheme performs excellent with low encoding-decoding complexity, its application to practical communication systems still has a long way to go since it is based on the noise-free feedback. In recent years, the SK-type schemes have been extensively studied for more practical feedback channel models, including quantized feedback channel [19], AWGN feedback channel [20], and feedback channel with arbitrary delay time [21]. However, note that in practical mobile communication scenarios, the feedback is often interrupted by obstacle and inter-cell handover etc., and this scenario is commonly modeled as intermittent feedback [22]. The optimal signaling strategies for the average-power-limited AWGN channel with intermittent feedback were investigated in [23,24], and [22,25] established bounds on the capacities of various channel models with intermittent feedback, which are based on random coding argument, and this implies that the coding schemes of [22,25] are non-constructive. Then it is natural to ask: is it possible to design efficient and low-complexity coding scheme for channels with intermittent feedback?

In this paper, we answer the aforementioned question by step-and-step extending the classical SK scheme to the following cases:

- We propose an SK-type schemes for the AWGN channel with noise-free intermittent feedback, and establish the relationship between the coding blocklength, the desired decoding error probability, and the achievable transmission rate of our proposed scheme.
- We further extend the above scheme to the noisy intermittent feedback cases.
- We analyze the secrecy levels of the above proposed schemes, and show that PLS can be achieved for some cases.

The remainder of this paper is organized as follows. Section 2 is about our results on the AWGN channel with noise-free/noisy intermittent feedback. Section 3 is about our results on the quasi-static fading channel with noise-free/noisy intermittent feedback. Security analysis of our proposed schemes is shown in Section 4. Section 5 concludes this paper and discusses future works.

## 2 The AWGN channel with intermittent feedback

### 2.1 Model formulation

**Fig 1:**
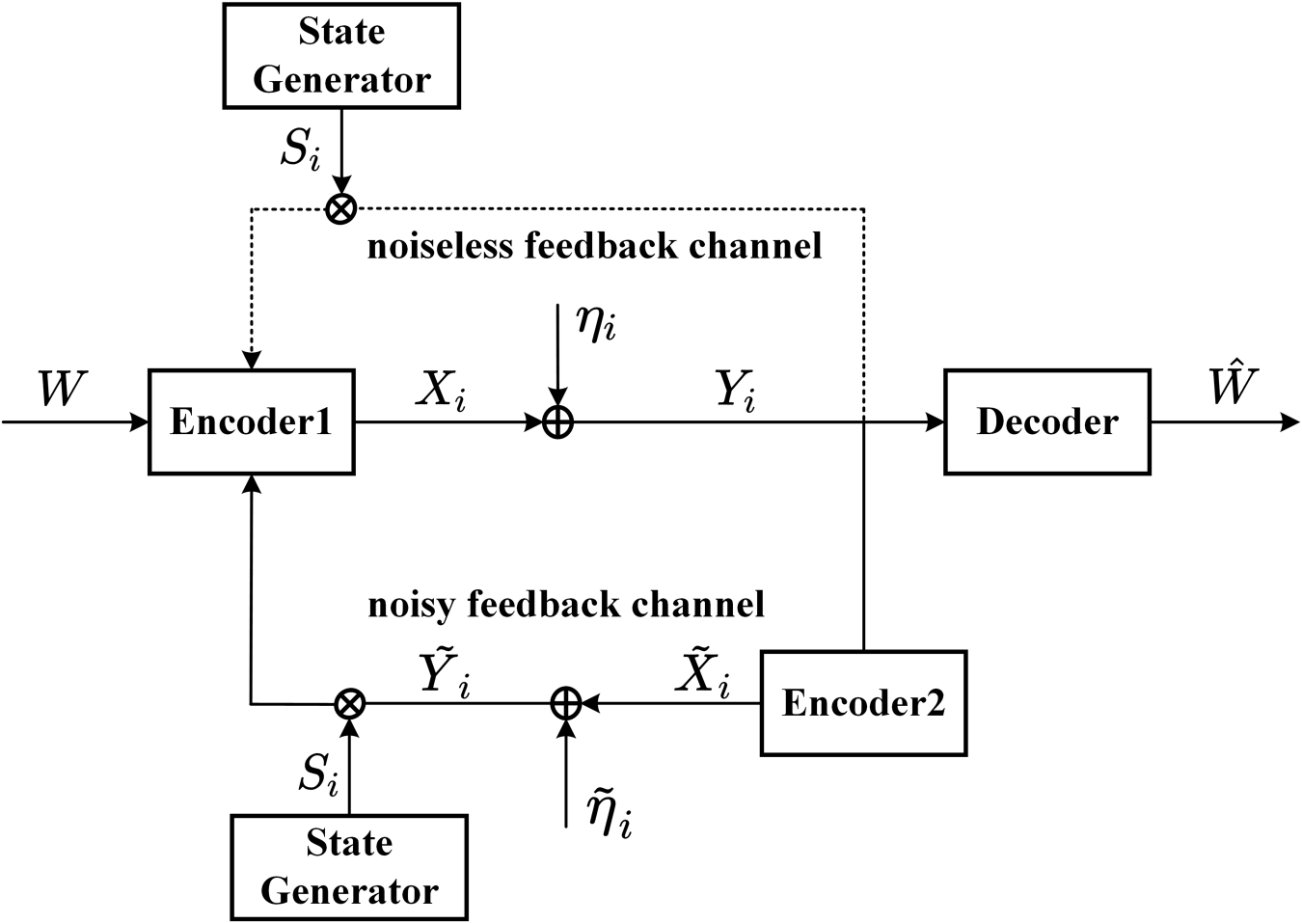
The AWGN channel with intermittent feedback.

**Channel**: Fig 1 shows the AWGN channel with intermittent feedback. For the feedfoward channel, the channel input-output is given by


$$\begin{array}{ll} & Y_{i}=X_{i}+\eta_{i},\;\;\;\;i\in \{1,2,...,N\}. \end{array}$$ (1)

For the *noisy* intermittent feedback, at time i∈{1,2,...,N}, the feedback channel input-output is given by


$$\begin{array}{ll} & {\tilde{Y}}_{i}=X_{i}+\eta_{i}. \end{array}$$ (2)

Here $\eta_{i}\sim 𝒩(0,\sigma^{2})$, ${\tilde{\eta }}_{i}\sim 𝒩(0,\sigma^{2})$ are identical independent distributed (i.i.d.) Gaussian noises and they are independent of each other.

**Intermittent feedback**: We introduce the state sequence


$$\begin{array}{l} S^{i}=(S_{1},S_{2},\cdot \cdot \cdot ,S_{i}), \end{array}$$ (3)

which denotes the Bernoulli process, and it is distributed i.i.d. over all time instants. Here note that *S*_*i*_ = 1 indicates that the transmitter perfectly obtains *Y*_*i*_, and *S*_*i*_ = 0 indicates that the transmitter fails to receive the receiver’s feedback at time instant *i*. Define


$$\begin{array}{l} \delta_{F}≜P\{S_{i}=0\}, \end{array}$$ (4)

for all i∈{1,2,...,N−1}, which is the probability that the transmitter fails to obtain the receiver’s feedback at time instant *i*.

**Encoder**:

- The input message *W* is uniformly distributed over the set $𝒲=\{1,2,...,2^{NR}\}$.
- For the noise-free feedback case, the encoder with output $X_{i}=f_{i}(W,S^{i-1},Y^{i-1})$ satisfies the average power constraint.

$$\begin{array}{l} \frac{1}{N}\sum_{i=1}^{N}E[{X_{i}}^{2}]\leq P, \end{array}$$ (5)

where $f_{i}(\cdot )$ is an encoding function of the transmitter at time index *i* (i∈{1,2,...,N}), $S^{i-1}=(S_{1},S_{2},...,S_{i-1})$ and $Y^{i-1}=(Y_{1},Y_{2},...,Y_{i-1})$. For simplification, define the feedforward signal-to-noise ratio as $SNR\overset{\text{def}}{=}\frac{P}{\sigma^{2}}$.

- For noisy feedback case, the encoder with output $X_{i}=f_{i}(W,S^{i-1},{\tilde{Y}}^{i-1})$ and ${\tilde{X}}_{i}={\tilde{f}}_{i}(Y^{i-1})$ satisfy the average power constraints

$$\begin{array}{l} \frac{1}{N}\sum_{i=1}^{N}E[{X_{i}}^{2}]\leq P, \end{array}$$ (6)

$$\begin{array}{l} \frac{1}{N-1}\sum_{i=1}^{N-1}E[{{\tilde{X}}_{i}}^{2}]\leq \tilde{P}, \end{array}$$ (7)

where $f_{i}(\cdot )$ and ${\tilde{f}}_{i}(\cdot )$ are encoding functions at time index *i* (i∈{1,2,...,N}), ${\tilde{Y}}^{i-1}=({\tilde{Y}}_{1},{\tilde{Y}}_{2},...,{\tilde{Y}}_{i-1})$, $S^{i-1}=(S_{1},S_{2},...,S_{i-1})$, and $Y^{i-1}=(Y_{1},Y_{2},...,Y_{i-1})$. For simplification, define the feedback signal-to-noise ratio as $\tilde{SNR}\overset{\text{def}}{=}\frac{\tilde{P}}{{\tilde{\sigma }}^{2}}$.

**Decoder**:

- The output of the decoder is $\hat{W}=\psi (Y^{N})$, where ψ is the decoding function of the receiver.
- The average decoding error probability is given by
  $$\begin{array}{l} P_{e}=\frac{1}{\left|𝒲\right|}\sum_{w\in 𝒲}Pr\{\psi (Y^{N})\neq w|w\;\;sent\}. \end{array}$$ (8)

**Achievable rate and capacity:**

The (N,ϵ)-rate *R* is achievable for given blocklength *N* and decoding error probability ϵ, there is a (N,|𝒲|,P)-code such that


$$\frac{\log|𝒲|}{N}\geq R-\epsilon ,\;\;\;\;P_{e}\leq \epsilon .$$ (9)

For the AWGN channel with noise-free intermittent feedback, the achievable rate is denoted by ${ℛ}^{f}(N,\epsilon )$, while for the noisy case, denoted by ${ℛ}^{nf}(N,\epsilon )$. Furthermore, the capacities ${𝒞}^{f}(N,\epsilon )$ and ${𝒞}^{nf}(N,\epsilon )$ are the maximum among all achievable rates defined above.

### 2.2 Main results and numerical examples

*Theorem 1*: For given coding blocklength *N* and decoding error probability ϵ, the lower bound ${ℛ}^{f}(N,\epsilon )$ on the capacity ${𝒞}^{f}(N,\epsilon )$ of the channel with intermittent noiseless feedback is given by


$$\begin{array}{ll} {𝒞}^{f}(N,\epsilon ) & \geq {ℛ}^{f}(N,\epsilon )\\ & =\frac{a_{N-1}\log\left(1+\frac{P}{\sigma^{2}}\right)}{2N}-\frac{\log\left\{2Q^{-1}(\frac{\epsilon }{2})\sqrt{\frac{\sigma^{2}}{12P}}\right\}}{N}, \end{array}$$ (10)

where $a_{N-1}\approx \left(1-\delta_{F})(N-1\right)$ for sufficiently large *N*.

*Proof*: See subsection 2.3.

*Theorem 2*: For given coding blocklength *N* and decoding error probability ϵ, the lower bound ${ℛ}^{nf}(N,\epsilon )$ on the capacity ${𝒞}^{nf}(N,\epsilon )$ of the channel with intermittent noisy feedback is given by


$$\begin{array}{ll} {𝒞}^{nf}\left(N,\epsilon \right) & \geq {ℛ}^{\;nf\;}(N,\epsilon )\\ & =\frac{1}{2N}\log\left(\frac{3\cdot SNR}{{\left[Q^{-1}(\frac{\epsilon }{4})\right]}^{2}}\cdot {\left(1+\frac{SNR}{\Psi_{1}\Psi_{2}}\right)}^{a_{N-1}}\right), \end{array}$$ (11)

$$\begin{array}{ll} & \Psi_{1}=1+L\cdot \frac{SNR}{S\tilde{N}R},\\ & \Psi_{2}=\frac{1}{1-L\cdot S\tilde{N}R^{-1}}, \end{array}$$ (12)

$$L=\frac{1}{3}{\left[Q^{-1}\left(\frac{\epsilon }{4a_{N-1}}\right)\right]}^{2},$$ (13)

where $a_{N-1}\approx \left(1-\delta_{F})(N-1\right)$ for sufficiently large *N*.

*Proof*: See subsection 2.3.

*Numerical result:*

**Fig 2:**
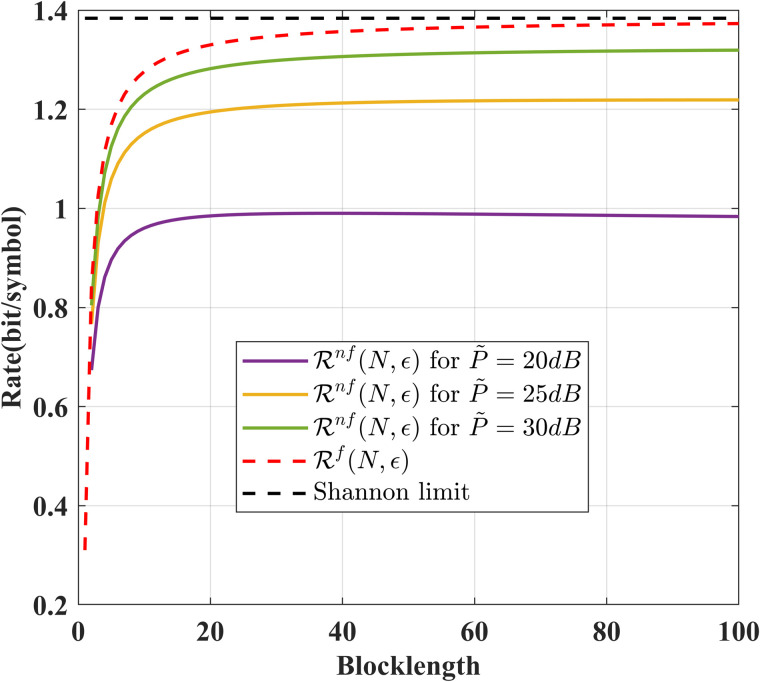
Comparison of ${ℛ}^{nf}(N,\epsilon )$ and ${ℛ}^{f}(N,\epsilon )$ for various *N* and *P* = 10 dB, $\delta_{F}=0.2$, $\epsilon =10^{-6}$, ${\tilde{\sigma }}^{2}=\sigma^{2}=1$.

Fig 2 compares the achievable rates of our schemes for noise-free and noisy feedback cases. From this figure, we conclude that the achievable rate for the noisy feedback case increases as the signal-to-noise ratio of the feedback channel increases, and it approaches its asymptotic value (the achievable rate for the noiseless feedback case) when the signal-to-noise ratio tends to infinity.

**Fig 3:**
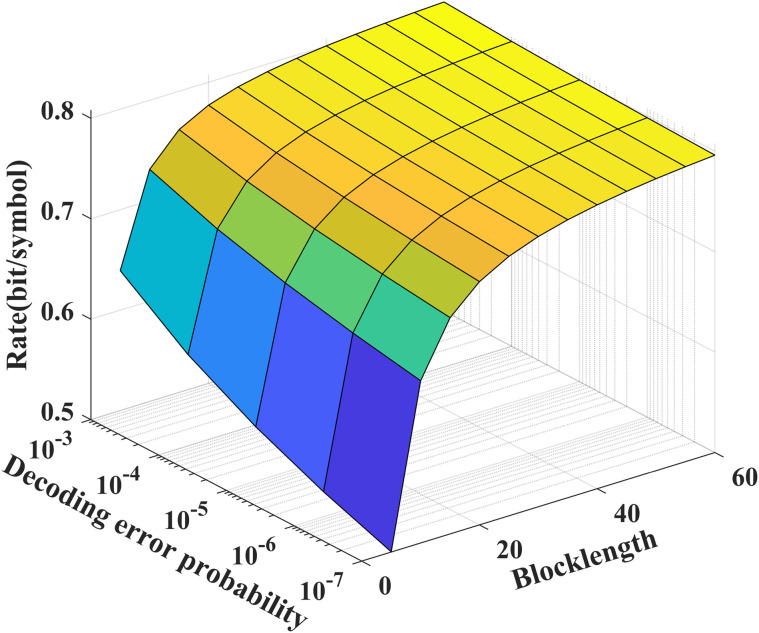
The relationship between ${ℛ}^{nf}(N,\epsilon )$, *P*_*e*_ and *N* for *P* = 5 dB, $\tilde{P}=20$ dB, $\delta_{F}=0.2$, and ${\tilde{\sigma }}^{2}=\sigma^{2}=1$.

**Fig 4:**
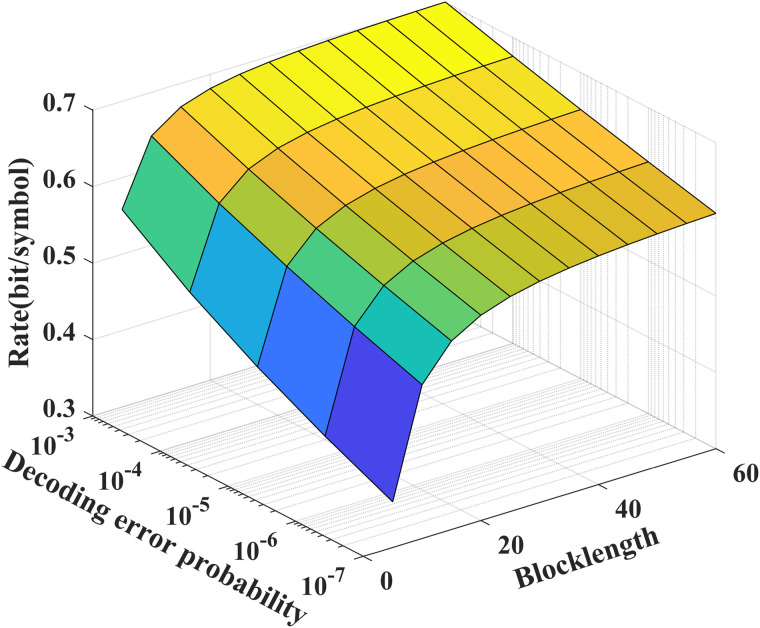
The relationship between ${ℛ}^{f}(N,\epsilon )$, *P*_*e*_ and *N* for *P* = 5 dB, $\delta_{F}=0.2$, and $\sigma^{2}=1$.

As shown in Figs 3 and 4, we see that to achieve a desired decoding error probability (10^−7^), the coding blocklength for the noise-free/noisy feedback case is about 50, which is significantly shorter than those of the LDPC and Polar codes. In addition, from Fig 4, we see that the feedback channel noise leads to the decreasing of the performance of our proposed scheme, e.g., for given *N* = 50, to achieve a desired decoding error probability (10^−7^), the transmission bits of the noisy feedback scheme is reduced to 75 percent of that of the noiseless feedback scheme.

**Fig 5:**
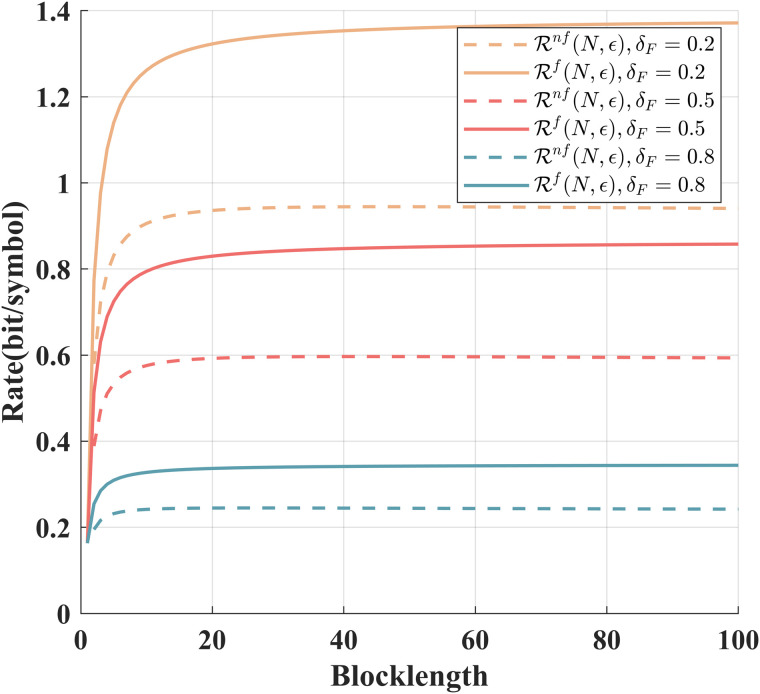
Comparison of ${ℛ}^{nf}(N,\epsilon )$ and ${ℛ}^{f}(N,\epsilon )$ under different feedback failure probabilities and *P* = 10 dB,$\tilde{P}=20$ dB, $\epsilon =10^{-6}$, ${\tilde{\sigma }}^{2}=\sigma^{2}=1$.

Fig 5 shows the relationship between the achievable rates of the scheme for the Gaussian channel with intermittent feedback under different $\delta_{F}$. When the coding blocklength is given, the achievable rate gradually decreases as $\delta_{F}$ increases. Under the same $\delta_{F}$, the achievable rates increase with the increase of the coding blocklength.

**Fig 6:**
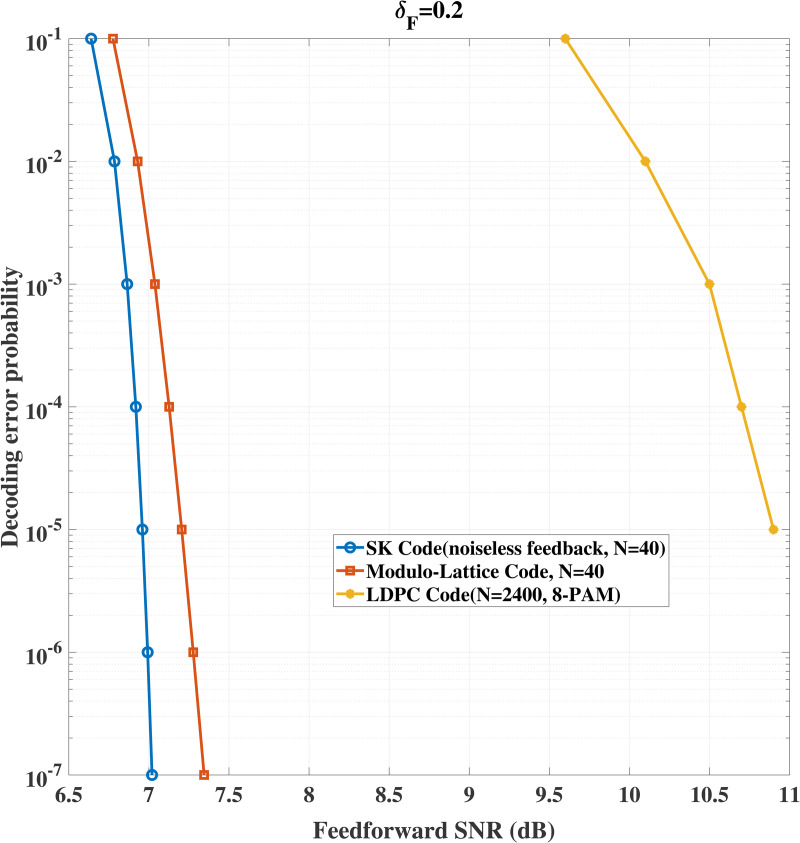
Performance comparison for $\delta_{F}=0.2$, *P* = 10 dB, ${\tilde{\sigma }}^{2}=\sigma^{2}=1$.

**Fig 7:**
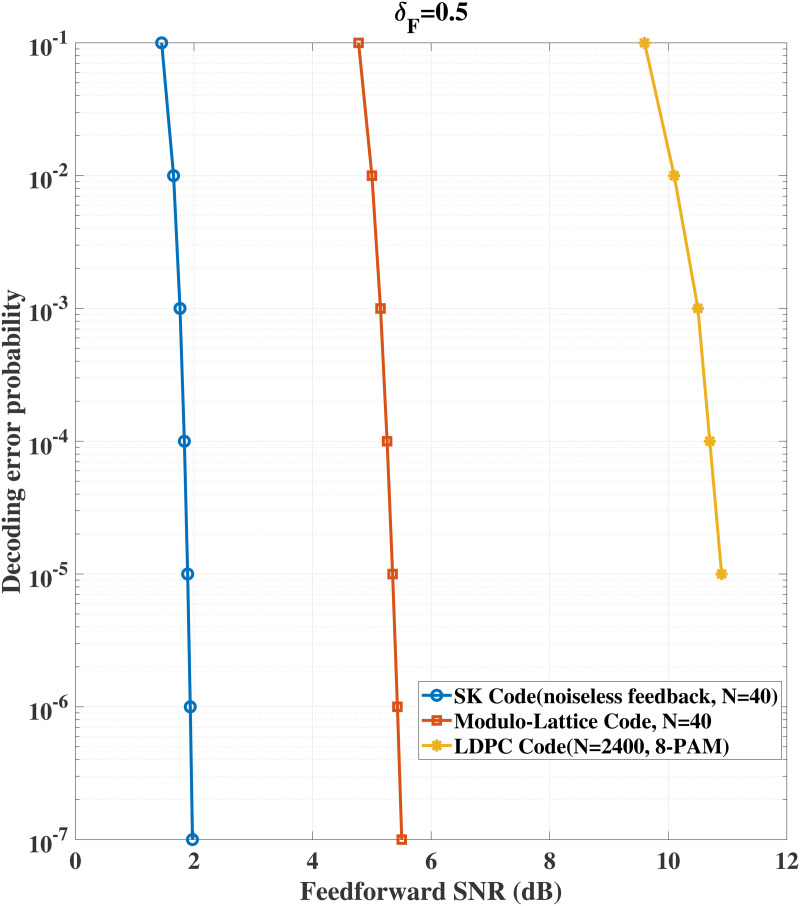
Performance comparison for $\delta_{F}=0.5$, *P* = 10 dB, ${\tilde{\sigma }}^{2}=\sigma^{2}=1$.

**Fig 8:**
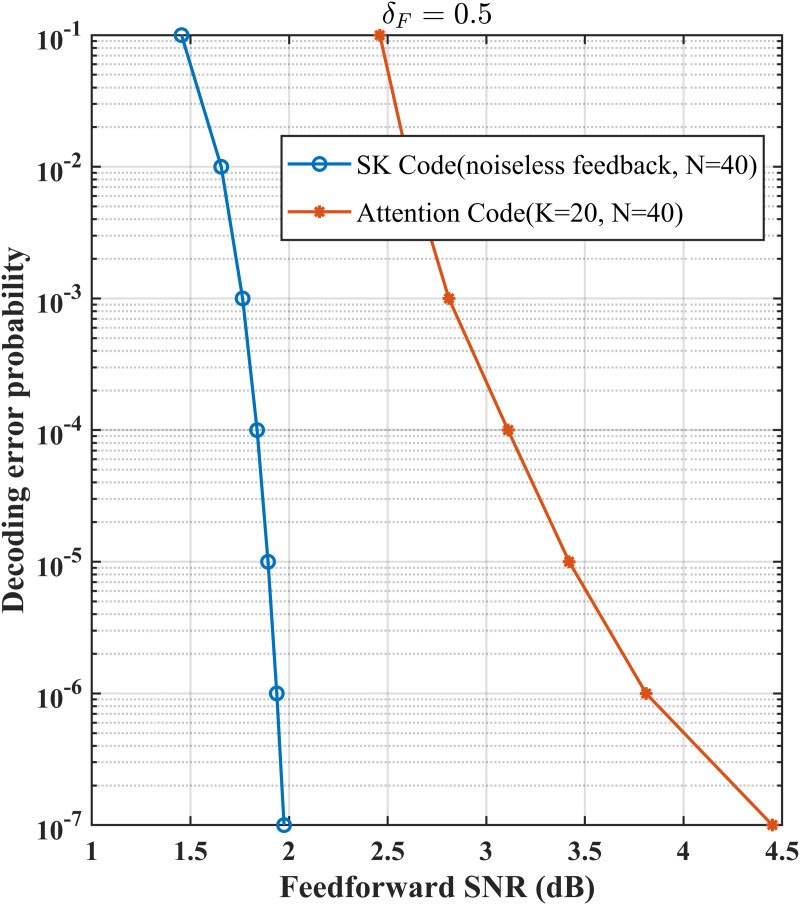
Performance comparison for $\delta_{F}=0.5$, *P* = 10 dB, $\sigma^{2}=1$.

We also provide performance comparison of our proposed schemes and LDPC code for the same model without using channel feedback. Specifically, Fig 6 shows that for $\delta_{F}=0.2$ and fixed transmission rate 1, to achieve a targeted decoding error probability, our proposed schemes require lower signal-to-noise ratio and significantly shorter coding blocklength comparing with LDPC code. Fig 7 shows that for $\delta_{F}=0.5$ and fixed transmission rate 0.5, the advantage of our schemes still holds. Besides this, comparison between the SK code and the AttentionCode [26] is given (see Fig 8). Fig 8 compares the decoding error probabilities of the SK code and AttentionCode under various channel qualities, and it is easy to see that the SK code performs better than the AttentionCode for some cases.

### 2.3 Proofs

**Proof of Theorem 1:**

Coding procedure:

Since *W* is uniformly distributed in 𝒲, θ is approximately uniformly distributed over the interval [−0.5,0.5] and its variance is approximately equal to $\frac{1}{12}$, i.e., $E[\theta^{2}]=\frac{1}{12}$. At time instant 1, the transmitter sends


$$X_{1}=\sqrt{12P}\theta .$$ (14)

At time instant *i* (2 ≤ *i* ≤ *N*), if *S*_*i*−1_ = 0, the transmitter repeatedly sends


$$X_{i}=X_{i-1}.$$ (15)

If *S*_*i*−1_ = 1, define $a_{i-1}=\pi (1|S^{i-1})$, where $\pi (1|S^{i-1})=\left|\left\{j:S_{j}=1\right\}\right|$ for j∈{1,···,i−1}. For example, if *S*^*i*−1^ = (0, 1, 1, 0, 0), $\pi (1|S^{i-1})=2$.

If *a*_*i*−1_ = 1, the receiver gets


$$Y_{i-1}=X_{i-1}+\eta_{i-1},$$ (16)

and sends it back to the transmitter, then computes his first estimation of θ by


$${\hat{\theta }}_{a_{i-1}}=\frac{Y_{i-1}}{\sqrt{12P}}=\theta +\frac{\eta_{i-1}}{\sqrt{12P}}=\theta +\varepsilon_{a_{i-1}},$$ (17)

where $\varepsilon_{a_{i-1}}={\hat{\theta }}_{a_{i-1}}-\theta =\frac{\eta_{i-1}}{\sqrt{12P}}$ is the estimation error, and its variance is $\alpha_{a_{i-1}}=Var(\varepsilon_{a_{i-1}})=\frac{\sigma^{2}}{12P}$. Thus, the transmitter sends the *i*-th time codeword *X*_*i*_ by


$$X_{i}=\sqrt{\frac{P}{\alpha_{a_{i-1}}}}\varepsilon_{a_{i-1}}.$$ (18)

If *a*_*i*−1_ > 1, the receiver gets $Y_{i-1}=X_{i-1}+\eta_{i-1}$ and sends it back to the transmitter. Then both the receiver and the transmitter calculate the estimation ${\hat{\theta }}_{a_{i-1}}$ by


$${\hat{\theta }}_{a_{i-1}}={\hat{\theta }}_{a_{i-2}}-\beta_{i-1}Y_{i-1}={\hat{\theta }}_{a_{i-2}}-{\hat{\varepsilon }}_{a_{i-2}},$$ (19)

where ${\hat{\varepsilon }}_{a_{i-2}}=\beta_{i-1}Y_{i-1}$, and $\beta_{i-1}$ is the Minimum Mean Squared Error (MMSE) estimation coefficient, which ensures that $\varepsilon_{a_{i-2}}$ is correctly estimated from *Y*_*i*−1_, and thus


$$\beta_{i-1}=\frac{E[\varepsilon_{a_{i-2}}Y_{i-1}]}{E[Y_{i-1}^{2}]}\overset{\text{(a)}}{=}\frac{\sqrt{P\alpha_{a_{i-2}}}}{P+\sigma^{2}},$$ (20)

where (*a*) follows from $Y_{i-1}=X_{i-1}+\eta_{i-1}$ and $X_{i-1}=\sqrt{\frac{P}{\alpha_{a_{i-2}}}}\varepsilon_{a_{i-2}}$. The updated estimation error is


$$\varepsilon_{a_{i-1}}={\hat{\theta }}_{a_{i-1}}-\theta =\varepsilon_{a_{i-2}}-\beta_{i-1}Y_{i-1},$$ (21)

and $\alpha_{a_{i-1}}=Var(\varepsilon_{a_{i-1}})$. Then the transmitter sends the *i*-th time codeword *X*_*i*_ by


$$X_{i}=\sqrt{\frac{P}{\alpha_{a_{i-1}}}}\varepsilon_{a_{i-1}}.$$ (22)

By calculation, it is not difficult to show that the general term of $\alpha_{a_{i-1}}$ is given by


$$\alpha_{a_{i-1}}=\frac{\sigma^{2}}{12P}(\frac{\sigma^{2}}{P+\sigma^{2}}{)}^{a_{i-1}-1}.$$ (23)

Decoding Error Probability Analysis: The receiver’s final estimation of θ is ${\hat{\theta }}_{a_{N}}=\theta +\varepsilon_{a_{N}}$, where *a*_*N*_ = *a*_*N*−1_ + 1, and the decoding error occurring at time instant *N* is defined as


$$E_{a_{N}}≜\{\varepsilon_{a_{N}}\notin [-\frac{1}{2\cdot 2^{N{ℛ}^{f}}},\frac{1}{2\cdot 2^{N{ℛ}^{f}}})\}.$$ (24)

Thus, the decoding error probability *P*_*e*_ is given by


$$\begin{array}{ll} P_{e} & =Pr\left\{\left|\varepsilon_{a_{N}}\right|\geq \frac{1}{2(\left|𝒲\right|-1)}\right\}\\ & \overset{\text{(a)}}{=}2Q\left(\frac{1}{2\cdot 2^{N{ℛ}^{f}}\cdot \sqrt{\alpha_{a_{N}}}}\right), \end{array}$$ (25)

where (*a*) follows from *Q*(*x*) is the tail of unit Gaussian distribution evaluated at *x*, and $\alpha_{a_{N}}$ denotes the variance of the receiver’s estimation error $\varepsilon_{a_{N}}$ at time instant *N*. From (25), we conclude that for obtaining the achievable rate *R*^*f*^, the variance $\alpha_{a_{N}}$ should be determined first, which is obtained as follows.

Statistical Analysis: First, from the coding procedure in the proof of Theorem 1, we calculate the receiver’s estimation error at each time instant as follows:

- Time 1: $\alpha_{a_{1}}=Var(\varepsilon_{a_{1}})=\frac{\sigma^{2}}{12P}$.
- Time *i* (2 ≤ *i* ≤ *N*): By (20) and (21), we have
  $$\begin{array}{ll} \alpha_{a_{i-1}} & =Var(\varepsilon_{a_{i-1}})=E{\left(\varepsilon_{a_{i-2}}-\frac{E(Y_{i-1}\varepsilon_{a_{i-2}})}{E(Y_{i-1}{)}^{2}}Y_{i-1}\right)}^{2}\\ & =E{\left(\varepsilon_{a_{i-2}}\right)}^{2}-2\frac{[E(Y_{i-1}\varepsilon_{a_{i-2}}){]}^{2}}{E(Y_{i-1}{)}^{2}}+\frac{[E(Y_{i-1}\varepsilon_{a_{i-2}}){]}^{2}}{E(Y_{i-1}{)}^{2}}\\ & =E{\left(\varepsilon_{a_{i-2}}\right)}^{2}-\frac{[E(Y_{i-1}\varepsilon_{a_{i-2}}){]}^{2}}{E(Y_{i-1}{)}^{2}}\\ & =\alpha_{a_{i-2}}-\frac{\alpha_{a_{i-2}}P}{P+\sigma^{2}}=\alpha_{a_{i-2}}\left\{\frac{\sigma^{2}}{P+\sigma^{2}}\right\}. \end{array}$$ (26)

By iteratively using (26), we have


$$\alpha_{a_{N}}=\frac{\sigma^{2}}{12P}{\left\{\frac{\sigma^{2}}{P+\sigma^{2}}\right\}}^{a_{N-1}-1},$$ (27)

which substituting into (25) and setting $P_{e}=\epsilon$ yields


$${ℛ}^{f}=\frac{a_{N-1}\log\left(1+\frac{P}{\sigma^{2}}\right)}{2N}-\frac{\log\left\{2Q^{-1}(\frac{\epsilon }{2})\sqrt{\frac{\sigma^{2}}{12P}}\right\}}{N}.$$ (28)

The proof is completed.

**Proof of Theorem 2:**

Coding procedure:

In the following, we introduce a shared dither random i.i.d. sequence $\{V_{i}{\}}_{i=1}^{N-1}$, which is perfectly known by both transmitter and receiver. $V_{i}\sim \text{Unif}([-d/2,d/2))$ and $d=\sqrt{12\tilde{P}}$. The sequence $\{V_{i}{\}}_{i=1}^{N-1}$ mutually independent of the noise sequences and the message, and it is used in the coding process. Then we describe the details of our coding scheme.

At time instant 1, the transmitter sends


$$X_{1}=\sqrt{12P}\theta .$$ (29)

At time instant *i* (2 ≤ *i* ≤ *N*), if *S*_*i*−1_ = 0, the transmitter repeatedly sends


$$X_{i}=X_{i-1}.$$ (30)

If *S*_*i*−1_ = 1 and *a*_*i*−1_ = 1, the receiver obtains $Y_{i-1}=X_{i-1}+\eta_{i-1}$ and calculates the first estimation of θ by


$${\hat{\theta }}_{a_{i-1}}=\frac{Y_{i-1}}{\sqrt{12P}}=\theta +\frac{\eta_{i-1}}{\sqrt{12P}}=\theta +\varepsilon_{a_{i-1}},$$ (31)

where $\varepsilon_{a_{i-1}}=\frac{\eta_{i-1}}{\sqrt{12P}}$ is the estimation error, and its variance is $\alpha_{a_{i-1}}=Var(\varepsilon_{a_{i-1}})=\frac{\sigma^{2}}{12P}$.

Then the receiver sends the estimation of θ to transmitter over the feedback channel by


$${\tilde{X}}_{i-1}={𝕄}_{d}[\gamma_{i-1}{\hat{\theta }}_{a_{i-1}}+V_{i-1}],$$ (32)

where $\gamma_{i-1}$ is the modulation coefficient to avoid modulo-aliasing. To eliminate the impact of the feedback channel noise, we apply the modulo-lattice operation ${𝕄}_{d}[\cdot ]$.

The transmitter obtains ${\tilde{Y}}_{i-1}={\tilde{X}}_{i-1}+{\tilde{\eta }}_{i-1}$, and then calculates a noisy version of the receiver’s estimation error by


$$\begin{array}{ll} {\tilde{\varepsilon }}_{a_{i-1}} & =\frac{1}{\gamma_{i-1}}{𝕄}_{d}[{\tilde{Y}}_{i-1}-\gamma_{i-1}\theta -V_{i-1}]\\ & =\frac{1}{\gamma_{i-1}}{𝕄}_{d}[\gamma_{i-1}\varepsilon_{a_{i-1}}+{\tilde{\eta }}_{i-1}], \end{array}$$ (33)

In the case when $\gamma_{i-1}\varepsilon_{a_{i-1}}+{\tilde{\eta }}_{i-1}\in [-\frac{d}{2},\frac{d}{2})$, which means the modulo-aliasing errors do not occur, we obtain


$$\begin{array}{ll} {\tilde{\varepsilon }}_{a_{i-1}} & =\frac{1}{\gamma_{i-1}}{𝕄}_{d}[\gamma_{i-1}\varepsilon_{a_{i-1}}+{\tilde{\eta }}_{i-1}]\\ & =\varepsilon_{a_{i-1}}+\frac{1}{\gamma_{i-1}}{\tilde{\eta }}_{i-1}, \end{array}$$ (34)

then the transmitter sends the *i*-th time codeword *X*_*i*_ by


$$X_{i}=\lambda_{i-1}\gamma_{i-1}{\tilde{\varepsilon }}_{a_{i-1}},$$ (35)

where $\lambda_{i-1}$ is set to satisfy input power constraint *P*.

if *a*_*i*−1_ > 1, the receiver obtains $Y_{i-1}=X_{i-1}+\eta_{i-1}$, and updates the estimation of θ by


$${\hat{\theta }}_{a_{i-1}}={\hat{\theta }}_{a_{i-2}}-\beta_{i-1}Y_{i-1},$$ (36)

where


$$\beta_{i-1}=\frac{E[\varepsilon_{a_{i-2}}Y_{i-1}]}{E[Y_{i-1}^{2}]}=\frac{E[\varepsilon_{a_{i-2}}(X_{i-1}+\eta_{i-1})]}{E[(X_{i-1}+\eta_{i-1}{)}^{2}]},$$ (37)

the estimation error at time instant *i* − 1 is


$$\varepsilon_{a_{i-1}}={\hat{\theta }}_{a_{i-1}}-\theta =\varepsilon_{a_{i-2}}-\beta_{i-1}Y_{i-1},$$ (38)

and the variance of $\varepsilon_{a_{i-1}}$ is denoted as $\alpha_{a_{i-1}}=Var(\varepsilon_{a_{i-1}})$.

Then the receiver sends the estimation of θ to the transmitter by


$${\tilde{X}}_{i-1}={𝕄}_{d}[\gamma_{i-1}{\hat{\theta }}_{a_{i-1}}+V_{i-1}],$$ (39)

the transmitter obtains ${\tilde{Y}}_{i-1}={\tilde{X}}_{i-1}+{\tilde{\eta }}_{i-1}$, and then calculate a noisy version of estimation error of the receiver by


$$\begin{array}{ll} {\tilde{\varepsilon }}_{a_{i-1}} & =\frac{1}{\gamma_{i-1}}{𝕄}_{d}[{\tilde{Y}}_{i-1}-\gamma_{i-1}\theta -V_{i-1}]\\ & =\frac{1}{\gamma_{i-1}}{𝕄}_{d}[\gamma_{i-1}\varepsilon_{a_{i-1}}+{\tilde{\eta }}_{i-1}], \end{array}$$ (40)

in the case when $\gamma_{i-1}\varepsilon_{a_{i-1}}+{\tilde{\eta }}_{i-1}\in [-\frac{d}{2},\frac{d}{2})$, we obtain ${\tilde{\varepsilon }}_{a_{i-1}}=\varepsilon_{a_{i-1}}+\frac{1}{\gamma_{i-1}}{\tilde{\eta }}_{i-1}$. The transmitter sends the *i*-th time codeword *X*_*i*_ by


$$X_{i}=\lambda_{i-1}\gamma_{i-1}{\tilde{\varepsilon }}_{a_{i-1}}.$$ (41)

Decoding Error Probability Analysis: The scheme is given in terms of the parameters $\gamma_{i-1}$, $\lambda_{i-1}$ and $\beta_{i-1}$ which dominate the transmission performance. The specific choices are described as follows.


$$\gamma_{i-1}=\sqrt{\frac{1}{\alpha_{a_{i-1}}}(\frac{\tilde{P}}{L}-{\tilde{\sigma }}^{2})},\lambda_{i-1}=\sqrt{L\frac{P}{\tilde{P}}},\;\beta_{i-1}=\frac{\sqrt{\alpha_{a_{i-2}}SNR\cdot (1-L\cdot {\tilde{SNR}}^{-1})}}{\sigma (1+SNR)},$$ (42)

where


$$\alpha_{a_{i-1}}=\frac{SNR^{-1}}{12}{\left(1+\frac{SNR}{\Psi_{1}\Psi_{2}}\right)}^{1-a_{i-1}},$$ (43)

$\Psi_{1}$ and $\Psi_{2}$ are defined in (12). The proof of (42) and (43) and the derivation of the achievable rate of our scheme are given in Appendix A. The proof is completed.

## 3 The quasi-static fading channel with intermittent feedback

### 3.1 Model formulation

**Fig 9:**
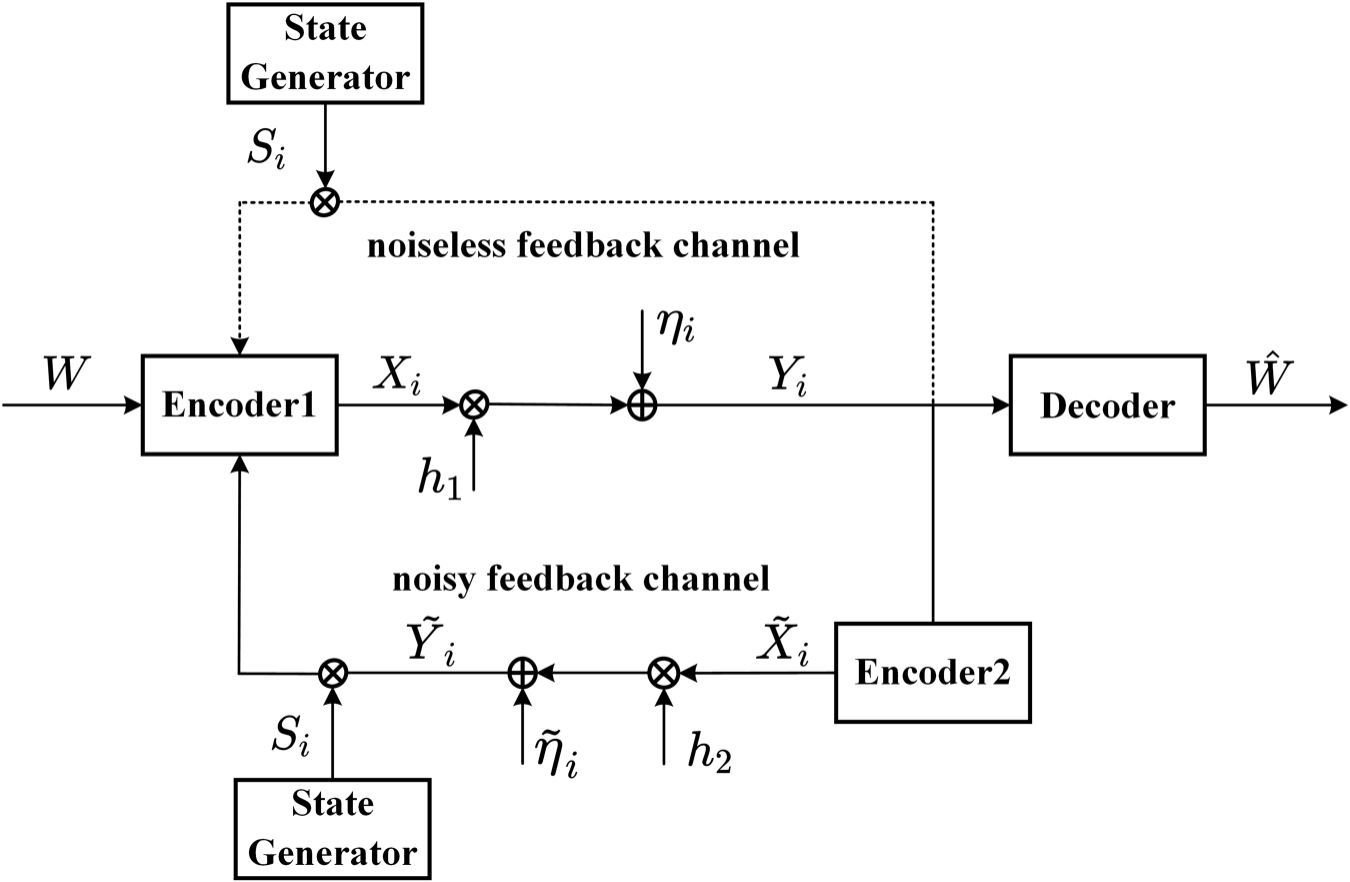
The quasi-static fading channel with intermittent feedback.

**Channel**: Fig 9 shows the quasi-static fading channel with intermittent feedback. For the feedfoward channel, the channel input-output is given by


$$\begin{array}{ll} & Y_{i}=h_{1}X_{i}+\eta_{i},\;\;\;\;i\in \{1,2,...,N\}. \end{array}$$ (44)

For the *noisy* intermittent feedback, at time i∈{1,2,...,N}, the feedback channel input-output is given by


$$\begin{array}{ll} & {\tilde{Y}}_{i}=h_{2}{\tilde{X}}_{i}+{\tilde{\eta }}_{i}. \end{array}$$ (45)

Here $\eta_{i}\sim CN(0,\sigma^{2})$ and ${\tilde{\eta }}_{i}∼CN(0,{\tilde{\sigma }}^{2})$ are i.i.d. circularly symmetric complex Gaussian noises and they are independent of each other.

The intermittent feedback is defined similar to that of the AWGN channel case, hence we omit its explanation here.

**Encoder**:

- The message *W* is uniformly distributed over the set $𝒲=\{1,2,...,2^{NR}\}$.
- For the scheme with noise-free feedback, the encoder with output $X_{i}=f_{i}(W,S^{i-1},Y^{i-1})$ satisfies the average power constraint
  $$\begin{array}{ll} & \frac{1}{N}\sum_{i=1}^{N}E[X_{i}X_{i}^{*}]\leq P, \end{array}$$ (46)
  where $f_{i}(\cdot )$ is an encoding function of the transmitter at time index *i* (i∈{1,2,...,N}), $S^{i-1}=(S_{1},S_{2},...,S_{i-1})$ and $Y^{i-1}=(Y_{1},Y_{2},...,Y_{i-1})$. For simplification, define the feedforward signal-to-noise ratio as $SNR\overset{\text{def}}{=}\frac{P}{\sigma^{2}}$.
- For the scheme with noisy feedback, the encoder with output $X_{i}=f_{i}(W,S^{i-1},{\tilde{Y}}^{i-1})$ and ${\tilde{X}}_{i}={\tilde{f}}_{i}(Y^{i-1})$ satisfy the average power constraints
  $$\begin{array}{ll} & \frac{1}{N}\sum_{i=1}^{N}E[X_{i}X_{i}^{*}]\leq P, \end{array}$$ (47)
  $$\begin{array}{ll} & \frac{1}{N-1}\sum_{i=1}^{N-1}E[{\tilde{X}}_{i}{\tilde{X}}_{i}^{*}]\leq \tilde{P}, \end{array}$$ (48)
  where $f_{i}(\cdot )$ and ${\tilde{f}}_{i}(\cdot )$ are encoding functions at time index *i* (i∈{1,2,...,N}), ${\tilde{Y}}^{i-1}=({\tilde{Y}}_{1},{\tilde{Y}}_{2},...,{\tilde{Y}}_{i-1})$, $S^{i-1}=(S_{1},S_{2},...,S_{i-1})$, and $Y^{i-1}=(Y_{1},Y_{2},...,Y_{i-1})$. For simplification, define the feedback signal-to-noise ratio as $\tilde{SNR}\overset{\text{def}}{=}\frac{\tilde{P}}{{\tilde{\sigma }}^{2}}$.

**Decoder**:

- The output of the decoder is $\hat{W}=\psi (Y^{N})$, where ψ is the decoding function of the receiver.
- The average decoding error probability is given by
  $$\begin{array}{ll} & P_{e}=\frac{1}{\left|𝒲\right|}\sum_{w\in 𝒲}Pr\{\psi (Y^{N})\neq w|w\;\;sent\}. \end{array}$$ (49)

**Achievable rate and capacity:**

For the fading channel with intermittent feedback, the achievable rates are denoted by ${ℛ}_{fc}^{f}(N,\epsilon )$ and ${ℛ}_{fc}^{nf}(N,\epsilon )$, and the capacities ${𝒞}_{fc}^{f}(N,\epsilon )$ and ${𝒞}_{fc}^{nf}(N,\epsilon )$ are the maximum among all achievable rates.

### 3.2 Main results and numerical examples

*Theorem 3*: For given coding blocklength *N* and decoding error probability ϵ, the lower bound ${ℛ}_{fc}^{f}(N,\epsilon )$ on the capacity ${𝒞}_{fc}^{f}(N,\epsilon )$ of the channel with intermittent noiseless feedback is given by


$$\begin{array}{ll} & {𝒞}_{fc}^{f}(N,\epsilon )\geq {ℛ}_{fc}^{f}(N,\epsilon )\\ & =\frac{a_{N-1}}{N}\log(1+SNR\cdot |h_{1}{|}^{2})-\frac{\log\left[(Q^{-1}(\frac{\epsilon }{4}){)}^{2}\frac{1}{3\cdot SNR\cdot |h_{1}{|}^{2}}\right]}{N}, \end{array}$$ (50)

where $a_{N-1}\approx \left(1-\delta_{F})(N-1\right)$ for sufficiently large *N*.

*Proof*: See subsection 3.3.

*Theorem 4*: For given coding blocklength *N* and decoding error probability ϵ, the lower bound ${ℛ}_{fc}^{nf}(N,\epsilon )$ on the capacity ${𝒞}_{fc}^{nf}(N,\epsilon )$ of the channel with intermittent noisy feedback is given by


$$\begin{array}{ll} & {𝒞}_{fc}^{nf}(N,\epsilon )\geq {ℛ}_{fc}^{nf}(N,\epsilon )\\ & =\frac{1}{N}\log\left(\frac{3\cdot SNR\cdot {\left|h_{1}\right|}^{2}}{{\left[Q^{-1}\left(\frac{\epsilon }{8}\right)\right]}^{2}}{\left(1+\frac{SNR\cdot {\left|h_{1}\right|}^{2}}{\psi_{1}\psi_{2}}\right)}^{a_{N-1}}\right), \end{array}$$ (51)

$$\psi_{1}=1+L\cdot SNR\cdot S\tilde{N}R^{-1}\cdot \frac{{\left|h_{1}\right|}^{2}}{{\left|h_{2}\right|}^{2}},$$ (52)

$$\psi_{2}={\left(1-\frac{L\cdot S\tilde{N}R^{-1}}{{\left|h_{2}\right|}^{2}}\right)}^{-1},$$ (53)

$$L=\frac{1}{3}{\left[Q^{-1}\left(\frac{\epsilon }{8a_{N-1}}\right)\right]}^{2},$$ (54)

where $a_{N-1}\approx \left(1-\delta_{F})(N-1\right)$ for sufficiently large *N*.

*Proof*: See subsection 3.3.

*Numerical result:*

**Fig 10:**
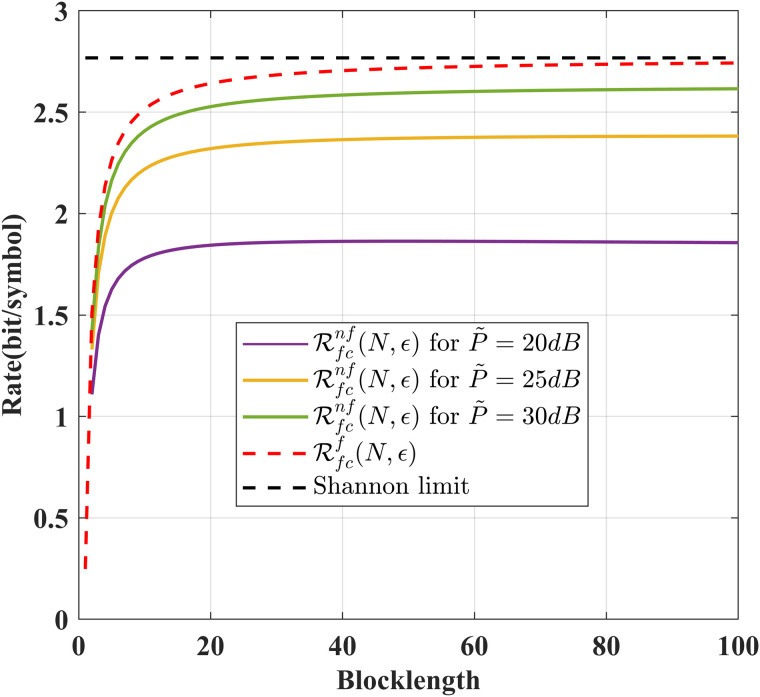
Comparison of ${ℛ}_{fc}^{nf}(N,\epsilon )$ and ${ℛ}_{fc}^{f}(N,\epsilon )$ for various *N* and *P* = 10 dB, $\delta_{F}=0.2$, $\epsilon =10^{-6}$, ${\tilde{\sigma }}^{2}=\sigma^{2}=1$, $h_{1},h_{2}\sim CN(0,1)$.

Fig 10 compares the achievable rates of fading channel schemes for noise-free and noisy feedback cases. From this figure, we conclude that the achievable rate for the noisy feedback case increases as the signal-to-noise ratio of the feedback channel increases, and it approaches the achievable rate for the noiseless feedback case when the signal-to-noise ratio tends to infinity.

**Fig 11:**
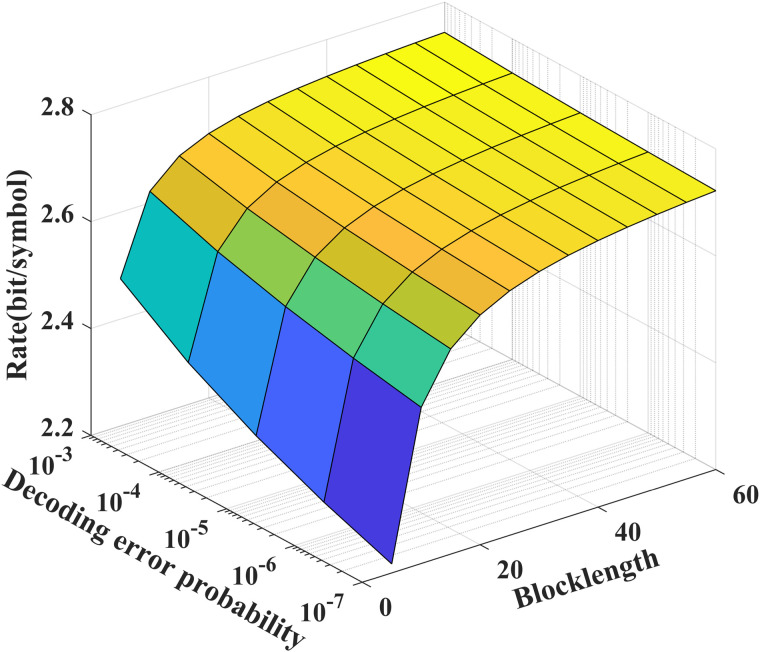
The relationship between ${ℛ}_{fc}^{f}(N,\epsilon )$, *P*_*e*_ and *N* for *P* = 10 dB, $\delta_{F}=0.2$, $h_{1},h_{2}\sim CN(0,1)$, and $\sigma^{2}=1$.

**Fig 12:**
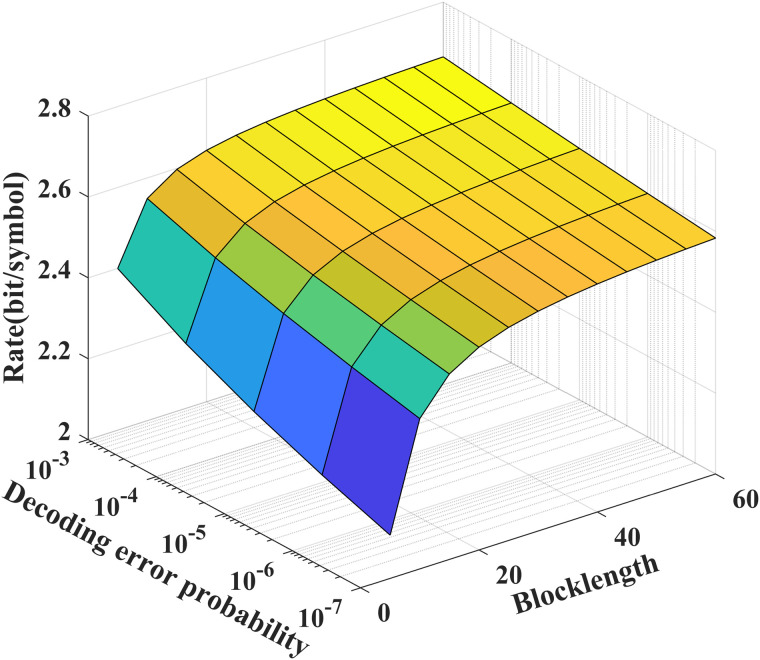
The relationship between ${ℛ}_{fc}^{nf}(N,\epsilon )$, *P*_*e*_ and *N* for *P* = 10 dB, $\tilde{P}=30$ dB, $\delta_{F}=0.2$, $h_{1},h_{2}\sim CN(0,1)$, and ${\tilde{\sigma }}^{2}=\sigma^{2}=1$.

As shown in Fig 11, we see that to achieve a desired decoding error probability (10^−7^), the coding blocklength for the noise-free/noisy feedback case is about 50, which is significantly shorter than those of the LDPC and Polar codes. In addition, from Fig 12, we see that the feedback channel noise leads to the decreasing of the performance of our proposed scheme.

**Fig 13:**
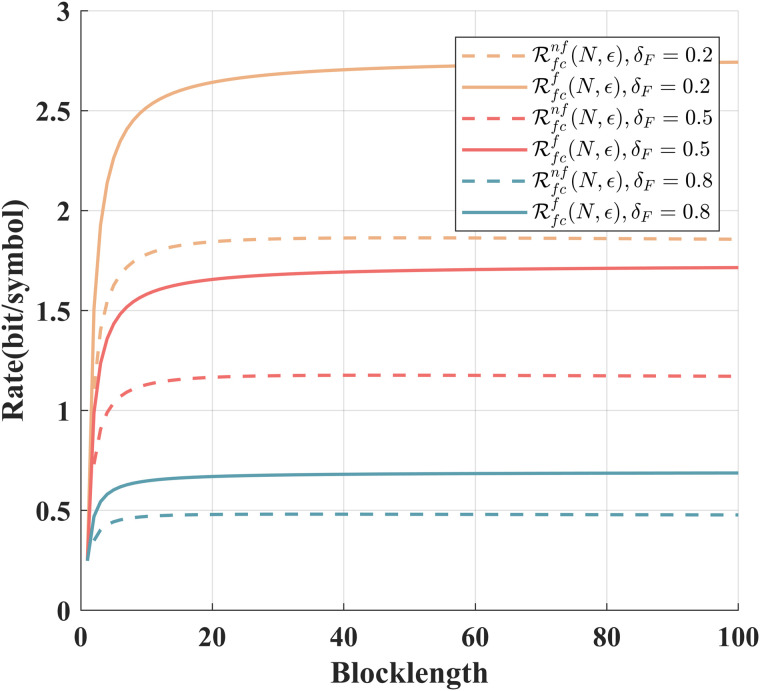
Comparison of ${ℛ}_{fc}^{nf}(N,\epsilon )$ and ${ℛ}_{fc}^{f}(N,\epsilon )$ under different feedback failure probabilities and *P* = 10 dB, $\tilde{P}=20$ dB, $\epsilon =10^{-6}$, ${\tilde{\sigma }}^{2}=\sigma^{2}=1$, $h_{1},h_{2}\sim CN(0,1)$.

Fig 13 shows the relationship between the achievable rates of the scheme for fading channel with intermittent feedback under different $\delta_{F}$. When the coding blocklength is given, the achievable rate gradually decreases as $\delta_{F}$ increases. Under the same $\delta_{F}$, the achievable rates increase with the increase of the coding blocklength.

### 3.3 Proofs

**Proof of Theorem 3:**

Coding procedure: At time instant *i*, since the elements in (44) are complex numbers, (44) can be re-written as


$$Y_{R,i}+jY_{I,i}=(h_{R,1}+jh_{I,1})(X_{R,i}+jX_{I,i})+\eta_{R,i}+j\eta_{I,i},$$ (55)

where $j=\sqrt{-1}$, *Y*_*R*,*i*_ = Re(*Y*_*i*_), *Y*_*I*,*i*_ = Im(*Y*_*i*_), $h_{R,1}=Re(h_{1})$, $h_{I,1}=Im(h_{1})$, $X_{R,i}=Re(X_{i})$, $X_{I,i}=Im(X_{i})$, $\eta_{R,i}=Re(\eta_{i})$, $\eta_{I,i}=Im(\eta_{i})$, where Re(·) and Im(·) denote the real and imaginary parts of a complex element, respectively. Here note that $E(X_{R,i}^{2})=P_{R}$ and $E(X_{I,i}^{2})=P_{I}$, where $P_{R}=P_{I}=\frac{1}{2}P$. Then convert (55) into real and imaginary parts


$$\begin{array}{ll} & Y_{R,i}=h_{R,1}X_{R,i}-h_{I,1}X_{I,i}+\eta_{R,i},\\ & Y_{I,i}=h_{R,1}X_{I,i}+h_{I,1}X_{R,i}+\eta_{I,i}, \end{array}$$ (56)

Then, we obtain


$$\begin{array}{ll} X_{R,i} & =\frac{h_{R,1}Y_{R,i}+h_{I,1}Y_{I,i}}{h_{R,1}^{2}+h_{I,1}^{2}}-\frac{h_{R,1}\eta_{R,i}+h_{I,1}\eta_{I,i}}{h_{R,1}^{2}+h_{I,1}^{2}}\\ X_{I,i} & =\frac{h_{R,1}Y_{I,i}-h_{I,1}Y_{R,i}}{h_{R,1}^{2}+h_{I,1}^{2}}-\frac{h_{R,1}\eta_{I,i}-h_{I,1}\eta_{R,i}}{h_{R,1}^{2}+h_{I,1}^{2}}, \end{array}$$ (57)

from (57), we define


$$\begin{array}{ll} & Y_{R,i}^{\prime }=\frac{h_{R,1}Y_{R,i}+h_{I,1}Y_{I,i}}{h_{R,1}^{2}+h_{I,1}^{2}},\;\eta_{R,i}^{\prime }=\frac{h_{R,1}\eta_{R,i}+h_{I,1}\eta_{I,i}}{h_{R,1}^{2}+h_{I,1}^{2}},\\ & Y_{I,i}^{\prime }=\frac{h_{R,1}Y_{I,i}-h_{I,1}Y_{R,i}}{h_{R,1}^{2}+h_{I,1}^{2}},\;\eta_{I,i}^{\prime }=\frac{h_{R,1}\eta_{I,i}-h_{I,1}\eta_{R,i}}{h_{R,1}^{2}+h_{I,1}^{2}}, \end{array}$$ (58)

thus (57) can be further expressed as


$$\begin{array}{ll} & X_{R,i}=Y_{R,i}^{\prime }-\eta_{R,i}^{\prime },\\ & X_{I,i}=Y_{I,i}^{\prime }-\eta_{I,i}^{\prime }, \end{array}$$ (59)

where $\eta_{R,i}^{\prime }\sim 𝒩(0,\frac{\sigma^{2}}{2|h_{1}{|}^{2}})$ and $\eta_{I,i}^{\prime }\sim 𝒩(0,\frac{\sigma^{2}}{2|h_{1}{|}^{2}})$. Hence, (59) is equivalent to (55), which indicates that the feedforward and feedback channels are divide into the two sub-channels.

Since *W* is divided two independent uniformly distributed sub-messages (*W*_*R*_, *W*_*I*_), where *W*_*R*_ and *W*_*I*_ take the value in the set ${𝒲}_{R}=\{1,2,...,2^{N{ℛ}_{R,fc}^{f}}\}$ and ${𝒲}_{I}=\{1,2,...,2^{N{ℛ}_{1,fc}^{f}}\}$, and we have ${ℛ}_{fc}^{f}={ℛ}_{R,fc}^{f}+{ℛ}_{I,fc}^{f}$. Divide the interval [−0.5,0.5] into ${ℛ}_{R,fc}^{f}$(${ℛ}_{I,fc}^{f}$) equally spaced sub-intervals, and the center of each sub-interval is mapped to a message value in *W*_*R*_(*W*_*I*_). Let $\theta_{R}$($\theta_{I}$) be the center of the sub-interval with respect to (w.r.t) the message *W*_*R*_(*W*_*I*_), and $E[\theta_{R}^{2}]=E[\theta_{I}^{2}]=\frac{1}{12}$.

At time instant 1, the transmitter sends


$$\begin{array}{ll} & X_{R,1}=\sqrt{12P_{R}}\theta_{R},\\ & X_{I,1}=\sqrt{12P_{I}}\theta_{I}. \end{array}$$ (60)

At time instant *i* (2 ≤ *i* ≤ *N*), if *S*_*i*−1_ = 0, the transmitter repeatedly sends


$$\begin{array}{ll} & X_{R,i}=X_{R,i-1},\\ & X_{I,1}=X_{I,i-1}. \end{array}$$ (61)

If *S*_*i*−1_ = 1, define $a_{i-1}=\pi (1|S^{i-1})$, where $\pi (1|S^{i-1})=\left|\left\{j:S_{j}=1\right\}\right|$ for j∈{1,···,i−1}. For example, if *S*^*i*−1^ = (0, 1, 1, 0, 0), $\pi (1|S^{i-1})=2$.

If *a*_*i*−1_ = 1, the receiver gets $Y_{i-1}=Y_{R,i-1}+jY_{I,i-1}$, and calculates the first equivalent output signal


$$\begin{array}{ll} & Y_{R,i-1}=X_{R,i-1}+\eta_{R,i-1}^{\prime }=\sqrt{P_{R}}\theta_{R}+\eta_{R,i-1}^{\prime },\\ & Y_{I,i-1}^{\prime }=X_{I,i-1}+\eta_{I,i-1}^{\prime }=\sqrt{P_{I}}\theta_{I}+\eta_{I,i-1}^{\prime }, \end{array}$$ (62)

and sends it back to the transmitter, then computes his first estimation of $\theta_{R}$ and $\theta_{I}$ by


$$\begin{array}{ll} & {\hat{\theta }}_{R,a_{i-1}}=\frac{Y_{R,i-1}^{\prime }}{\sqrt{P_{R}}}=\theta_{R}+\frac{\eta_{R,i-1}^{\prime }}{\sqrt{P_{R}}}=\theta_{R}+\varepsilon_{R,a_{i-1}},\\ & {\hat{\theta }}_{I,a_{i-1}}=\frac{Y_{I,i-1}^{\prime }}{\sqrt{P_{I}}}=\theta_{I}+\frac{\eta_{I,i-1}^{\prime }}{\sqrt{P_{I}}}=\theta_{I}+\varepsilon_{I,a_{i-1}}, \end{array}$$ (63)

then the transmitter can get the estimation error of the receiver


$$\begin{array}{ll} & \varepsilon_{R,a_{i-1}}={\hat{\theta }}_{R,a_{i-1}}-\theta_{R}=\frac{\eta_{R,i-1}^{\prime }}{\sqrt{P_{R}}},\\ & \varepsilon_{I,a_{i-1}}={\hat{\theta }}_{I,a_{i-1}}-\theta_{I}=\frac{\eta_{I,i-1}^{\prime }}{\sqrt{P_{I}}}, \end{array}$$ (64)

Thus, the transmitter sends the *i*-th time codeword by


$$\begin{array}{ll} & X_{R,i}=\sqrt{\frac{P_{R}}{\alpha_{R,a_{i-1}}}}\varepsilon_{R,a_{i-1}},\\ & X_{I,i}=\sqrt{\frac{P_{I}}{\alpha_{I,a_{i-1}}}}\varepsilon_{I,a_{i-1}}. \end{array}$$ (65)

If *a*_*i*−1_ > 1, the receiver gets $Y_{i-1}=Y_{R,i-1}+jY_{I,i-1}$ and sends it back to the transmitter. Then both the receiver and the transmitter calculate the estimation by


$$\begin{array}{ll} & {\hat{\theta }}_{R,a_{i-1}}={\hat{\theta }}_{R,a_{i-2}}-\beta_{R,i-1}Y_{R,i-1}^{\prime },\\ & {\hat{\theta }}_{I,a_{i-1}}={\hat{\theta }}_{I,a_{i-2}}-\beta_{I,i-1}Y_{I,i-1}^{\prime }, \end{array}$$ (66)

where


$$\begin{array}{ll} & \beta_{R,i-1}=\frac{E(Y_{R,i-1}^{{\;}^{\prime }}\varepsilon_{R,a_{i-2}})}{E(Y_{R,i-1}^{\prime }{)}^{2}},\\ & \beta_{I,i-1}=\frac{E(Y_{I,i-1}^{{\;}^{\prime }}\varepsilon_{I,a_{i-2}})}{E(Y_{I,i-1}^{\prime }{)}^{2}}, \end{array}$$ (67)

the updated estimation error are


$$\begin{array}{ll} & \varepsilon_{R,a_{i-1}}=\varepsilon_{R,a_{i-2}}-\beta_{R,i-1}Y_{R,i-1}^{\prime },\\ & \varepsilon_{I,a_{i-1}}=\varepsilon_{I,a_{i-2}}-\beta_{I,i-1}Y_{I,i-1}^{\prime }, \end{array}$$ (68)

then the transmitter sends the *i*-th time codeword by


$$\begin{array}{ll} & X_{R,i}=\sqrt{\frac{P_{R}}{\alpha_{R,a_{i-1}}}}\varepsilon_{R,a_{i-1}},\\ & X_{I,i}=\sqrt{\frac{P_{I}}{\alpha_{I,a_{i-1}}}}\varepsilon_{I,a_{i-1}}. \end{array}$$ (69)

Statistical Analysis: By calculation, it is not difficult to show that the general term of $\alpha_{R,a_{i-1}}$ is given by


$$\begin{array}{ll} \alpha_{R,a_{i-1}} & =Var(\varepsilon_{R,a_{i-1}})\\ & =E{\left(\varepsilon_{R,a_{i-2}}-\frac{E(Y_{i-1}^{\prime }\varepsilon_{R,a_{i-2}})}{E(Y_{i-1}^{\prime }{)}^{2}}Y_{i-1}^{\prime }\right)}^{2}\\ & =E{\left(\varepsilon_{R,a_{i-2}}\right)}^{2}-\frac{[E(Y_{i-1}^{\prime }\varepsilon_{R,a_{i-2}}){]}^{2}}{E(Y_{i-1}^{\prime }{)}^{2}}\\ & =\alpha_{R,a_{i-2}}-\frac{\alpha_{R,a_{i-2}}P_{R}}{P_{R}+\frac{\sigma^{2}}{2|h_{1}{|}^{2}}}\\ & =\alpha_{R,a_{i-2}}\left\{\frac{\sigma^{2}}{P|h_{1}{|}^{2}+\sigma^{2}}\right\}\\ & =\frac{\sigma^{2}}{12P}{\left\{\frac{\sigma^{2}}{P|h_{1}{|}^{2}+\sigma^{2}}\right\}}^{a_{i-1}-1} \end{array}$$ (70)

Performance Analysis:

Since *W* is divided into two independent uniformly distributed sub-messages (*W*_*R*_, *W*_*I*_), we define the decoding error probability of (*W*_*R*_, *W*_*I*_) as *P*_*e*,*R*_ and *P*_*e*,*I*_. The decoding error probability analysis process is the same for the real part and the imaginary part, and the corresponding decoding error probability of the real part is analyzed below. The receiver’s final estimation of $\theta_{R}$ is ${\hat{\theta }}_{R,a_{N}}=\theta_{R}+\varepsilon_{R,a_{N}}$, where $a_{N}=a_{N-1}+1$, and the decoding error probability ${\hat{\theta }}_{R,a_{N}}$ occurring at time instant *N* is defined as


$$\begin{array}{ll} P_{e,R} & =\mathrm{Pr}\left\{\varepsilon_{R,a_{N}}\notin \left[-\frac{1}{2\cdot 2^{NR_{R,fc}^{f}}},\frac{1}{2\cdot 2^{NR_{R,fc}^{f}}}\right]\right\}\\ & =2Q\left(\frac{1}{2\cdot 2^{NR_{R,fc}^{f}}}\cdot \frac{1}{\sqrt{\alpha_{R,a_{N}}}}\right). \end{array}$$ (71)

For given coding blocklength *N* and decoding error probability $P_{e,R}=\frac{\epsilon }{2}$, substituting (70) into (71), we can get


$${ℛ}_{R,fc}^{f}=\frac{a_{N-1}}{2N}\log(1+\frac{|h_{1}{|}^{2}P}{\sigma^{2}})-\frac{\log\left\{2Q^{-1}(\frac{\epsilon }{4})\sqrt{\frac{\sigma^{2}}{12P|h_{1}{|}^{2}}}\right\}}{N}),$$ (72)

similarly, we can get ${ℛ}_{R,fc}^{f}$


$${ℛ}_{I,fc}^{f}=\frac{a_{N-1}}{2N}\log(1+\frac{|h_{1}{|}^{2}P}{\sigma^{2}})-\frac{\log\left\{2Q^{-1}(\frac{\epsilon }{4})\sqrt{\frac{\sigma^{2}}{12P|h_{1}{|}^{2}}}\right\}}{N}.$$ (73)

From (72) and (73), the achievable rate of the fading channel with intermittent noise-free feedback is given by


$$\begin{array}{ll} {ℛ}_{fc}^{f} & ={ℛ}_{R,fc}^{f}+{ℛ}_{I,fc}^{f}\\ & =\frac{a_{N-1}}{N}\log(1+SNR\cdot |h_{1}{|}^{2})-\frac{\log\left[[Q^{-1}(\frac{\epsilon }{4}){]}^{2}\frac{1}{3\cdot SNR\cdot |h_{1}{|}^{2}}\right]}{N}, \end{array}$$ (74)

where $a_{N-1}\approx \left(1-\delta_{F})(N-1\right)$ for sufficiently large *N*. The proof is completed.

**Proof of Theorem 4:**

At time instant *i*, since the elements in (44) are complex numbers, (44) can be re-written as


$$\begin{array}{ll} & Y_{R,i}+jY_{I,i}=(h_{R,1}+jh_{I,1})(X_{R,i}+jX_{I,i})+\eta_{R,i}+j\eta_{I,i},\\ & {\tilde{Y}}_{R,i}+j{\tilde{Y}}_{I,i}=(h_{R,2}+jh_{I,2})({\tilde{X}}_{R,i}+j{\tilde{X}}_{I,i})+{\tilde{\eta }}_{R,i}+j{\tilde{\eta }}_{I,i}, \end{array}$$ (75)

where $j=\sqrt{-1}$, *Y*_*R*,*i*_ = Re(*Y*_*i*_), $Y_{I,i}=Im(Y_{i})$, $h_{R,1}=Re(h_{1})$, $h_{I,1}=Im(h_{1})$, $X_{R,i}=Re(X_{i})$, $X_{I,i}=Im(X_{i})$, $\eta_{R,i}=Re(\eta_{i})$, $\eta_{I,i}=Im(\eta_{i})$, ${\tilde{Y}}_{R,i}=Re({\tilde{Y}}_{i})$, ${\tilde{Y}}_{I,i}=Im({\tilde{Y}}_{i})$, $h_{R,2}=Re(h_{2})$, $h_{I,2}=Im(h_{2})$, ${\tilde{X}}_{R,i}=Re({\tilde{X}}_{i})$, ${\tilde{X}}_{I,i}=Im({\tilde{X}}_{i})$, ${\tilde{\eta }}_{R,i}=Re({\tilde{\eta }}_{i})$, ${\tilde{\eta }}_{I,i}=Im({\tilde{\eta }}_{i})$, where Re(·) and Im(·) denote the real and imaginary parts of a complex element, respectively. Here note that $E(X_{R,i}^{2})=P_{R}$, $E(X_{I,i}^{2})=P_{I}$, $E({\tilde{X}}_{R,i}^{2})={\tilde{P}}_{R}$, and $E({\tilde{X}}_{I,i}^{2})={\tilde{P}}_{I}$ where $P_{R}=P_{I}=\frac{1}{2}P$ and ${\tilde{P}}_{R}={\tilde{P}}_{I}=\frac{1}{2}\tilde{P}$. Then convert (75) into real and imaginary parts


$$\begin{array}{ll} & Y_{R,i}=h_{R,1}X_{R,i}-h_{I,1}X_{I,i}+\eta_{R,i},\\ & Y_{I,i}=h_{R,1}X_{I,i}+h_{I,1}X_{R,i}+\eta_{I,i},\\ & {\tilde{Y}}_{R,i}=h_{R,2}{\tilde{X}}_{R,i}-h_{I,2}{\tilde{X}}_{I,i}+{\tilde{\eta }}_{R,i},\\ & {\tilde{Y}}_{I,i}=h_{R,2}{\tilde{X}}_{I,i}+h_{I,2}{\tilde{X}}_{R,i}+{\tilde{\eta }}_{I,i}, \end{array}$$ (76)

where $\eta_{{\;}_{R,i}}\sim CN(0,\sigma_{{\;}_{R}}^{2}=\frac{\sigma^{2}}{2})$, $\eta_{{\;}_{I,i}}\sim CN(0,\sigma_{{\;}_{I}}^{2}=\frac{\sigma^{2}}{2})$, ${\tilde{\eta }}_{{\;}_{R,i}}\sim CN(0,{\tilde{\sigma }}_{{\;}_{R}}^{2}=\frac{{\tilde{\sigma }}^{2}}{2})$, and ${\tilde{\eta }}_{I,i}\sim CN(0,{\tilde{\sigma }}_{I}^{2}=\frac{{\tilde{\sigma }}^{2}}{2})$. Then, we obtain


$$\begin{array}{ll} & X_{R,i}=\frac{h_{R,1}Y_{R,i}+h_{I,1}Y_{I,i}}{h_{R,1}^{2}+h_{I,1}^{2}}-\frac{h_{R,1}\eta_{R,i}+h_{I,1}\eta_{I,i}}{h_{R,1}^{2}+h_{I,1}^{2}},\\ & X_{I,i}=\frac{h_{R,1}Y_{I,i}-h_{I,1}Y_{R,i}}{h_{R,1}^{2}+h_{I,1}^{2}}-\frac{h_{R,1}\eta_{I,i}-h_{I,1}\eta_{R,i}}{h_{R,1}^{2}+h_{I,1}^{2}},\\ & {\tilde{X}}_{R,i}=\frac{h_{R,2}{\tilde{Y}}_{R,i}+h_{I,2}{\tilde{Y}}_{I,i}}{h_{R,2}^{2}+h_{I,2}^{2}}-\frac{h_{R,2}{\tilde{\eta }}_{R,i}+h_{I,2}{\tilde{\eta }}_{I,i}}{h_{R,2}^{2}+h_{I,2}^{2}},\\ & {\tilde{X}}_{I,i}=\frac{h_{R,2}{\tilde{Y}}_{I,i}-h_{I,2}{\tilde{Y}}_{R,i}}{h_{R,2}^{2}+h_{I,2}^{2}}-\frac{h_{R,2}{\tilde{\eta }}_{I,i}-h_{I,2}{\tilde{\eta }}_{R,i}}{h_{R,2}^{2}+h_{I,2}^{2}}, \end{array}$$ (77)

from (77), we define


$$\begin{array}{ll} & Y_{R,i}^{\prime }=\frac{h_{R,1}Y_{R,i}+h_{I,1}Y_{I,i}}{h_{R,1}^{2}+h_{I,1}^{2}},\;\eta_{R,i}^{\prime }=\frac{h_{R,1}\eta_{R,i}+h_{I,1}\eta_{I,i}}{h_{R,1}^{2}+h_{I,1}^{2}},\\ & Y_{I,i}^{\prime }=\frac{h_{R,1}Y_{I,i}-h_{I,1}Y_{R,i}}{h_{R,1}^{2}+h_{I,1}^{2}},\;\eta_{I,i}^{\prime }=\frac{h_{R,1}\eta_{I,i}-h_{I,1}\eta_{R,i}}{h_{R,1}^{2}+h_{I,1}^{2}},\\ & {\tilde{Y}}_{R,i}^{\prime }=\frac{h_{R,2}Y_{R,i}+h_{I,2}{\tilde{Y}}_{I,i}}{h_{R,2}^{2}+h_{I,2}^{2}},\;{\tilde{\eta }}_{R,i}^{\prime }=\frac{h_{R,2}{\tilde{\eta }}_{R,i}+h_{I,2}{\tilde{\eta }}_{I,i}}{h_{R,2}^{2}+h_{I,2}^{2}},\\ & {\tilde{Y}}_{I,i}^{\prime }=\frac{h_{R,2}Y_{I,i}-h_{I,2}{\tilde{Y}}_{R,i}}{h_{R,2}^{2}+h_{I,2}^{2}},\;{\tilde{\eta }}_{I,i}^{\prime }=\frac{h_{R,2}{\tilde{\eta }}_{I,i}-h_{I,2}{\tilde{\eta }}_{R,i}}{h_{R,2}^{2}+h_{I,2}^{2}}, \end{array}$$ (78)

thus (77) can be further expressed as


$$X_{R,i}=Y_{R,i}^{\prime }-\eta_{R,i}^{\prime },\;X_{I,i}=Y_{I,i}^{\prime }-\eta_{I,i}^{\prime },\;{\tilde{X}}_{R,i}={\tilde{Y}}_{R,i}^{\prime }-{\tilde{\eta }}_{R,i}^{\prime },\;{\tilde{X}}_{I,i}={\tilde{Y}}_{I,i}^{\prime }-{\tilde{\eta }}_{I,i}^{\prime },$$ (79)

where $\eta_{R,i}^{\prime }\sim 𝒩(0,\frac{\sigma^{2}}{2|h_{1}{|}^{2}})$, $\eta_{I,i}^{\prime }\sim 𝒩(0,\frac{\sigma^{2}}{2|h_{1}{|}^{2}})$, ${\tilde{\eta }}_{R,i}^{\prime }\sim 𝒩(0,\frac{{\tilde{\sigma }}^{2}}{2|h_{2}{|}^{2}})$ and ${\tilde{\eta }}_{I,i}^{\prime }\sim 𝒩(0,\frac{{\tilde{\sigma }}^{2}}{2|h_{2}{|}^{2}})$. Hence, (79) is equivalent to (75), which indicates that the feedforward and feedback channels are divide into the two sub-channels.

Since *W* is divided two independent uniformly distributed sub-messages (*W*_*R*_, *W*_*I*_), where *W*_*R*_ and *W*_*I*_ take the value in the set ${𝒲}_{R}=\{1,2,...,2^{N{ℛ}_{R,fc}^{nf}}\}$ and ${𝒲}_{I}=\{1,2,...,2^{N{ℛ}_{I,fc}^{nf}}\}$, and we have ${ℛ}_{fc}^{nf}={ℛ}_{R,fc}^{nf}+{ℛ}_{I,fc}^{nf}$. The interval $\left[-\sqrt{3},\sqrt{3}\right]$ divides into ${ℛ}_{R,fc}^{nf}$(${ℛ}_{I,fc}^{nf}$) sub-intervals, and maps *W*_*R*_(*W*_*I*_) to the center of sub-interval. Since *W*_*R*_(*W*_*I*_) is uniformly distributed in ${𝒲}_{R}$(${𝒲}_{I}$), $\theta_{R}$($\theta_{I}$) is approximately uniformly distributed over the interval $\left[-\sqrt{3},\sqrt{3}\right]$ and its variance is approximately equal to 1, i.e., $E[\theta_{R}^{2}]=E[\theta_{I}^{2}]=1$.

In the following, we also introduce shared dither random i.i.d. sequence $V_{R}^{N-1}=(V_{R,1},...,V_{R,N-1})$ and $V_{I}^{N-1}=(V_{I,1},...,V_{I,N-1})$, $V_{R}\sim Unif[-\frac{d_{R}}{2},\frac{d_{R}}{2}]$ and $V_{I}\sim Unif[-\frac{d_{I}}{2},\frac{d_{I}}{2}]$, where $d_{R}=\sqrt{12{\tilde{P}}_{R}}$, $d_{I}=\sqrt{12{\tilde{P}}_{I}}$. The sequence $V_{R}^{N-1}$ and $V_{I}^{N-1}$ mutually independent of the noise sequences and the message, and they are involved in the following iterative encoding procedure, which ensures that the encoded signals satisfy the transmission power constraints. Then we describe the details of our coding scheme.

At time instant 1, the transmitter sends


$$\begin{array}{ll} & X_{R,1}=\sqrt{P_{R}}\theta_{R},\\ & X_{I,1}=\sqrt{P_{I}}\theta_{I}. \end{array}$$ (80)

At time instant *i* (2 ≤ *i* ≤ *N*), if *S*_*i*−1_ = 0, the transmitter repeatedly sends


$$\begin{array}{ll} & X_{R,i}=X_{R,i-1},\\ & X_{I,1}=X_{I,i-1}. \end{array}$$ (81)

if *S*_*i*−1_ = 1 and *a*_*i*−1_ = 1, the receiver obtains $Y_{i-1}=Y_{R,i-1}+jY_{I,i-1}$ and calculates the first equivalent output signal


$$\begin{array}{ll} & Y_{R,i-1}^{\prime }=X_{R,i-1}+\eta_{R,i-1}^{\prime }=\sqrt{P_{R}}\theta_{R}+\eta_{R,i-1}^{\prime },\\ & Y_{I,i-1}^{\prime }=X_{I,i-1}+\eta_{I,i-1}^{\prime }=\sqrt{P_{I}}\theta_{I}+\eta_{I,i-1}^{\prime }, \end{array}$$ (82)

and sends it back to the transmitter, then computes his first estimation of $\theta_{R}$ and $\theta_{I}$ by


$$\begin{array}{ll} & {\hat{\theta }}_{R,a_{i-1}}=\frac{Y_{R,i-1}^{\prime }}{\sqrt{P_{R}}}=\theta_{R}+\frac{\eta_{R,i-1}^{\prime }}{\sqrt{P_{R}}}=\theta_{R}+\varepsilon_{R,a_{i-1}},\\ & {\hat{\theta }}_{I,a_{i-1}}=\frac{Y_{I,i-1}^{\prime }}{\sqrt{P_{I}}}=\theta_{I}+\frac{\eta_{I,i-1}^{\prime }}{\sqrt{P_{I}}}=\theta_{I}+\varepsilon_{I,a_{i-1}}, \end{array}$$ (83)

then the transmitter can get the estimation error of the receiver


$$\begin{array}{ll} & \varepsilon_{R,a_{i-1}}={\hat{\theta }}_{R,a_{i-1}}-\theta_{R}=\frac{\eta_{R,i-1}^{\prime }}{\sqrt{P_{R}}},\\ & \varepsilon_{I,a_{i-1}}={\hat{\theta }}_{I,a_{i-1}}-\theta_{I}=\frac{\eta_{I,i-1}^{\prime }}{\sqrt{P_{I}}}, \end{array}$$ (84)

where


$$\begin{array}{ll} & \alpha_{R,a_{i-1}}=Var(\varepsilon_{R,a_{i-1}})=\frac{\sigma^{2}}{2|h_{1}{|}^{2}P_{R}},\\ & \alpha_{I,a_{i-1}}=Var(\varepsilon_{I,a_{i-1}})=\frac{\sigma^{2}}{2|h_{1}{|}^{2}P_{I}}, \end{array}$$ (85)

Then the receiver sends the estimation of $\theta_{R}$ and $\theta_{I}$ to transmitter over the feedback channel by


$$\begin{array}{ll} & {\tilde{X}}_{R,i-1}={𝕄}_{d}\left[\gamma_{R,i-1}{\hat{\theta }}_{R,a_{i-1}}+V_{R,i-1}\right],\\ & {\tilde{X}}_{I,i-1}={𝕄}_{d}\left[\gamma_{I,i-1}{\hat{\theta }}_{I,a_{i-1}}+V_{I,i-1}\right], \end{array}$$ (86)

where $\gamma_{R,i-1}$ and $\gamma_{I,i-1}$ are the modulation coefficient to avoid modulo-aliasing. To eliminate the impact of the feedback channel noise, we apply the modulo-lattice operation ${𝕄}_{d}[\cdot ]$.

The transmitter obtains


$$\begin{array}{ll} & {\tilde{Y}}_{R,i-1}^{{\;}^{\prime }}={\tilde{X}}_{R,i-1}+{\tilde{\eta }}_{R,i-1}^{{\;}^{\prime }},\\ & {\tilde{Y}}_{I,i-1}^{{\;}^{\prime }}={\tilde{X}}_{I,i-1}+{\tilde{\eta }}_{I,i-1}^{{\;}^{\prime }}, \end{array}$$ (87)

and then calculates a noisy version of the receiver’s estimation error by


$$\begin{array}{ll} {\tilde{\varepsilon }}_{R,a_{i-1}} & =\frac{1}{\gamma_{R,i-1}}{𝕄}_{d}\left[{\tilde{Y}}_{R,i-1}^{{\;}^{\prime }}-\gamma_{R,i-1}\theta_{R}-V_{R,i-1}\right]\\ & =\frac{1}{\gamma_{R,i-1}}{𝕄}_{d}\left[\gamma_{R,i-1}\varepsilon_{R,a_{i-1}}+{\tilde{\eta }}_{R,i-1}^{{\;}^{\prime }}\right],\\ {\tilde{\varepsilon }}_{I,a_{i-1}} & =\frac{1}{\gamma_{I,i-1}}{𝕄}_{d}\left[{\tilde{Y}}_{I,i-1}^{{\;}^{\prime }}-\gamma_{I,i-1}\theta_{I}-V_{I,i-1}\right]\\ & =\frac{1}{\gamma_{I,i-1}}{𝕄}_{d}\left[\gamma_{I,i-1}\varepsilon_{I,a_{i-1}}+{\tilde{\eta }}_{I,i-1}^{{\;}^{\prime }}\right], \end{array}$$ (88)

In the case when $\gamma_{R,i-1}\varepsilon_{R,a_{i-1}}+{\tilde{\eta }}_{R,i-1}^{\prime }\in [\begin{array}{l} -\frac{d_{R}}{2},\frac{d_{R}}{2} \end{array}]$ and $\gamma_{I,i-1}\varepsilon_{I,a_{i-1}}+{\tilde{\eta }}_{I,i-1}^{\prime }\in [\begin{array}{l} -\frac{d_{I}}{2},\frac{d_{I}}{2} \end{array}]$, which means the modulo-aliasing errors do not occur, we obtain


$$\begin{array}{ll} & {\tilde{\varepsilon }}_{R,a_{i-1}}=\varepsilon_{R,a_{i-1}}+\frac{1}{\gamma_{R,i-1}}{\tilde{\eta }}_{R,i-1}^{\prime },\\ & {\tilde{\varepsilon }}_{I,a_{i-1}}=\varepsilon_{I,a_{i-1}}+\frac{1}{\gamma_{I,i-1}}{\tilde{\eta }}_{I,i-1}^{\prime }, \end{array}$$ (89)

then the transmitter sends the *i*-th time codeword by


$$\begin{array}{ll} & X_{R,i}=\lambda_{R,i-1}\gamma_{R,i-1}{\tilde{\varepsilon }}_{R,a_{i-1}},\\ & X_{I,i}=\lambda_{I,i-1}\gamma_{I,i-1}{\tilde{\varepsilon }}_{I,a_{i-1}}, \end{array}$$ (90)

where $\lambda_{R,i-1}$ and $\lambda_{I,i-1}$ are set to satisfy input power constraint *P*_*R*_ and *P*_*I*_.

If *a*_*i*−1_ > 1, the receiver obtains $Y_{i-1}=Y_{R,i-1}+jY_{I,i-1}$, and updates the estimation of $\theta_{R}$ and $\theta_{I}$ by


$$\begin{array}{ll} & {\hat{\theta }}_{R,a_{i-1}}={\hat{\theta }}_{R,a_{i-2}}-\beta_{R,i-1}Y_{R,i-1}^{\prime },\\ & {\hat{\theta }}_{I,a_{i-1}}={\hat{\theta }}_{I,a_{i-2}}-\beta_{I,i-1}Y_{I,i-1}^{\prime }, \end{array}$$ (91)

where


$$\begin{array}{ll} & \beta_{R,i-1}=\frac{E(Y_{R,i-1}^{\prime }\varepsilon_{R,a_{i-2}})}{E(Y_{R,i-1}^{\prime }{)}^{2}},\\ & \beta_{I,i-1}=\frac{E(Y_{I,i-1}^{\prime }\varepsilon_{I,a_{i-2}})}{E(Y_{I,i-1}^{\prime }{)}^{2}}, \end{array}$$ (92)

the estimation error at time instant *i* − 1 is


$$\begin{array}{ll} & \varepsilon_{R,a_{i-1}}=\varepsilon_{R,a_{i-2}}-\beta_{R,i-1}Y_{R,i-1}^{{\;}^{\prime }},\\ & \varepsilon_{I,a_{i-1}}=\varepsilon_{I,a_{i-2}}-\beta_{I,i-1}Y_{I,i-1}^{{\;}^{\prime }}, \end{array}$$ (93)

and the variance of $\varepsilon_{R,a_{i-1}}$ and $\varepsilon_{I,a_{i-1}}$ is denoted as $\alpha_{R,a_{i-1}}=Var(\varepsilon_{R,a_{i-1}})$ and $\alpha_{I,a_{i-1}}=Var(\varepsilon_{I,a_{i-1}})$.

Then the receiver sends the estimation of $\theta_{R}$ and $\theta_{I}$ to the transmitter by


$$\begin{array}{ll} & {\tilde{X}}_{R,i-1}={𝕄}_{d}\left[\gamma_{R,i-1}{\hat{\theta }}_{R,a_{i-1}}+V_{R,i-1}\right],\\ & {\tilde{X}}_{I,i-1}={𝕄}_{d}\left[\gamma_{I,i-1}{\hat{\theta }}_{I,a_{i-1}}+V_{I,i-1}\right], \end{array}$$ (94)

the transmitter obtains ${\tilde{Y}}_{R,i-1}^{\prime }={\tilde{X}}_{R,i-1}+{\tilde{\eta }}_{R,i-1}^{\prime }$ and ${\tilde{Y}}_{I,i-1}^{\prime }={\tilde{X}}_{I,i-1}+{\tilde{\eta }}_{I,i-1}^{\prime }$, and then calculate a noisy version of estimation error of the receiver by


$$\begin{array}{ll} {\tilde{\varepsilon }}_{R,a_{i-1}} & =\frac{1}{\gamma_{R,i-1}}{𝕄}_{d}\left[Y_{R,i-1}^{\prime }-\gamma_{R,i-1}\theta_{R}-V_{R,i-1}\right]\\ & =\frac{1}{\gamma_{R,i-1}}{𝕄}_{d}\left[\gamma_{R,i-1}\varepsilon_{R,a_{i-1}}+{\tilde{\eta }}_{R,i-1}^{\prime }\right],\\ {\tilde{\varepsilon }}_{I,a_{i-1}} & =\frac{1}{\gamma_{I,i-1}}{𝕄}_{d}\left[Y_{I,i-1}^{\prime }-\gamma_{I,i-1}\theta_{I}-V_{I,i-1}\right]\\ & =\frac{1}{\gamma_{I,i-1}}{𝕄}_{d}\left[\gamma_{I,i-1}\varepsilon_{I,a_{i-1}}+{\tilde{\eta }}_{I,i-1}^{\prime }\right], \end{array}$$ (95)

in the case when $\gamma_{R,i-1}\varepsilon_{R,a_{i-1}}+{\tilde{\eta }}_{R,i-1}^{\prime }\in [\begin{array}{l} -\frac{d_{R}}{2},\frac{d_{R}}{2} \end{array}]$ and $\gamma_{I,i-1}\varepsilon_{I,a_{i-1}}+{\tilde{\eta }}_{I,i-1}^{\prime }\in [\begin{array}{l} -\frac{d_{I}}{2},\frac{d_{I}}{2} \end{array}]$. The transmitter sends the *i*-th time codeword by


$$\begin{array}{ll} & X_{R,i}=\lambda_{R,i-1}\gamma_{R,i-1}{\tilde{\varepsilon }}_{R,a_{i-1}},\\ & X_{I,i}=\lambda_{I,i-1}\gamma_{I,i-1}{\tilde{\varepsilon }}_{I,a_{i-1}}. \end{array}$$ (96)

Performance Analysis: In our proposed scheme, we define the parameters $\gamma_{R,i-1}$, $\lambda_{R,i-1}$ and $\beta_{R,i-1}$ as follows, and the transmission performance of our scheme is determined by these parameters.


$$\gamma_{R,i-1}=\sqrt{\frac{1}{\alpha_{R,a_{i-1}}}\left(\frac{\tilde{P}}{2L}-\frac{{\tilde{\sigma }}^{2}}{2\left(|h_{2}{|}^{2}\right)}\right)},$$ (97)

$$\lambda_{R,i-1}=\sqrt{L\frac{P}{\tilde{P}}},$$ (98)

$$\beta_{{\;}_{R,i-1}}=\frac{\sqrt{2\alpha_{R,a_{I-2}}}}{\sigma }\cdot \frac{\sqrt{SNR\left(1-L\cdot S\tilde{N}R^{-1}{\left(|h_{2}{|}^{2}\right)}^{-1}\right)}}{SNR+{\left(|h_{2}{|}^{2}\right)}^{-1}},$$ (99)

where


$$\alpha_{R,a_{i-1}}=SNR^{-1}\cdot {\left(|h_{1}{|}^{2}\right)}^{-1}{\left(1+\frac{SNR\cdot \left(|h_{1}{|}^{2}\right)}{\psi_{1}\psi_{2}}\right)}^{1-a_{i-1}}$$ (100)

$\psi_{1}$ and $\psi_{2}$ are defined in (52) and (53). The proof of $\gamma_{R,i-1}$, $\lambda_{R,i-1}$, $\beta_{R,i-1}$ and the derivation of the achievable rate of our scheme are given in Appendix B. The proof is completed.

## 4 Security analysis of our schemes

### 4.1 The fading wiretap channel with intermittent noise-free feedback

**Fig 14:**
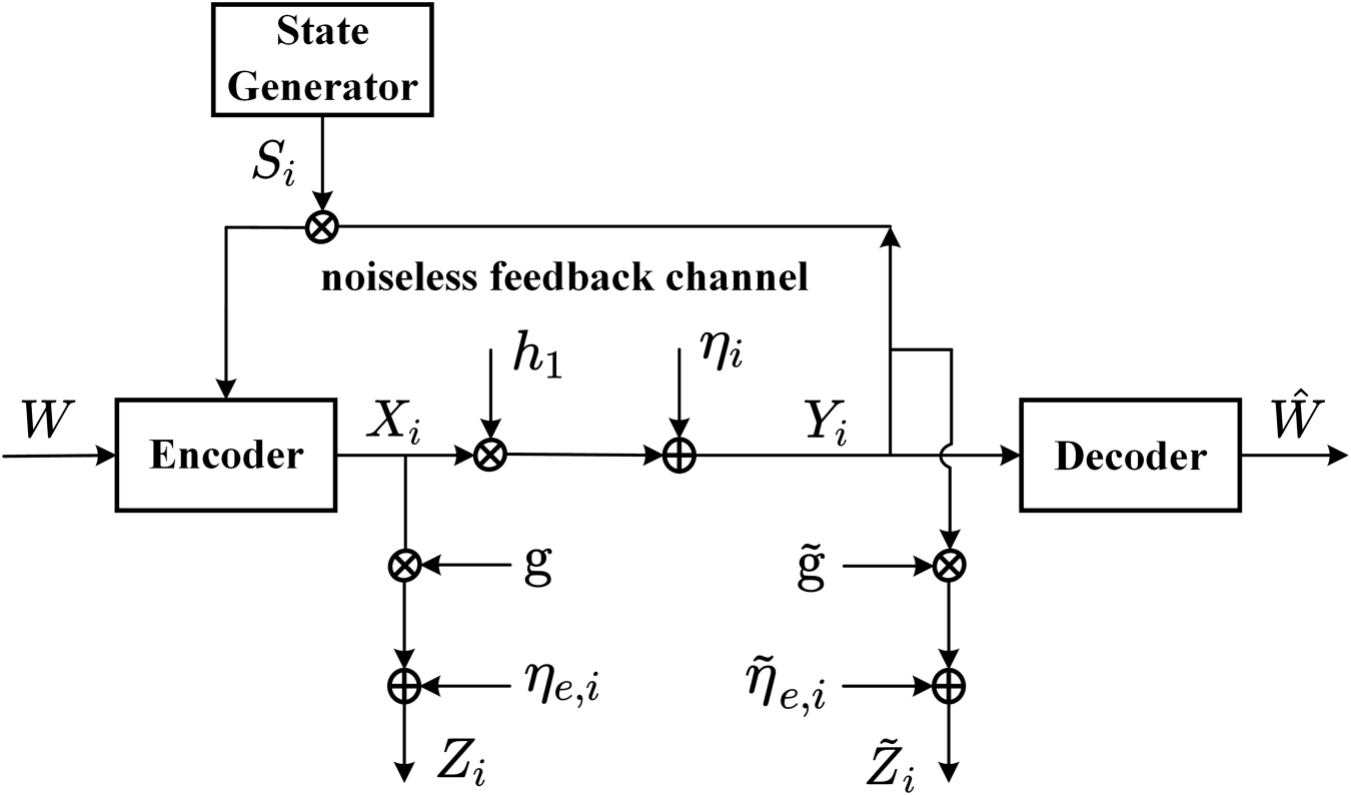
The fading wiretap channel with intermittent noiseless feedback.

In this subsection, we analyze the secrecy level of our scheme for the fading wiretap channel with intermittent noise-free feedback, see Fig 14.

In Fig 14, the wiretap channel is given by


$$\begin{array}{l} \begin{array}{l} \begin{array}{ll} & Z_{i}=gX_{i}+\eta_{e,i},1\leq i\leq N\\ & {\tilde{Z}}_{i}=\tilde{g}Y_{i}+{\tilde{\eta }}_{e,i},1\leq i\leq N-1, \end{array} \end{array} \end{array}$$ (101)

where $X_{i}\in \mathbb{C}$, $Y_{i}\in \mathbb{C}$ are the input and output of the feedforward channel at time instant *i*. The $\eta_{i}$, $\eta_{e,i}$ and ${\tilde{\eta }}_{e,i}$ are i.i.d as $CN(0,\sigma^{2})$, $CN(0,{\sigma_{e}}^{2})$ and $CN(0,{\tilde{\sigma_{e}}}^{2})$, respectively. $g,\tilde{g}\in \mathbb{C}$ are the fading coefficients of the eavesdropping channel.

**Definition:**

The uncertainty of the eavesdropper (also called the secrecy level) is defined as


$$\begin{array}{l} \Delta =\frac{H(W|Z_{1}^{N},{\tilde{Z}}_{1}^{N-1},h_{1},g,\tilde{g})}{H(W)},\;0\leq \Delta \leq 1, \end{array}$$ (102)

where $Z_{1}^{N}=(Z_{1},Z_{2},...,Z_{N})$ and ${\tilde{Z}}_{1}^{N-1}=({\tilde{Z}}_{1},{\tilde{Z}}_{2},...,{\tilde{Z}}_{N-1})$, Δ=1 corresponds to perfect secrecy, which follows from the same definition in [27].

For the fading eavesdropping channel scheme with noise-free intermittent feedback, the codeword sent at the previous instant should be re-sent at the next instant due to the failure of feedback, hence if the feedback failure occurs, it has an impact on the secrecy level, to this end, we define until the time instant


$$\begin{array}{l} k(1\leq k\leq \delta_{F}(N-1)), \end{array}$$ (103)

the codeword containing the θ is successfully sent and feedback.

*Theorem 5*: For given coding blocklength *N* and decoding error probability ϵ, the lower bound on the security level of the fading channel with noise-free intermittent feedback is given by


$$\begin{array}{l} \Delta_{I}\geq 1-\frac{k\{\log(1+\frac{P|g{|}^{2}}{\sigma_{e}^{2}})+\log(1+\frac{P|\tilde{g}h_{{\;}_{1}}{|}^{2}}{{\tilde{\sigma }}_{e}^{2}})\}}{N\cdot {ℛ}_{fc}^{f}(N,\epsilon )}, \end{array}$$ (104)

where $k\log(1+\frac{P|g{|}^{2}}{\sigma_{e}^{2}})$ and $k\log(1+\frac{P|\tilde{g}h_{1}{|}^{2}}{{\tilde{\sigma }}_{e}^{2}})$ represent the information leaked through the feedforward channel and the feedback channel, respectively.

**Proof.** From (102), we can obtain


$$\begin{array}{l} \begin{array}{l} \begin{array}{ll} \Delta_{I} & =\frac{H(W|Z_{1}^{N},{\tilde{Z}}_{1}^{N-1},h_{1},g,\tilde{g})}{H(W)}\\ & \overset{\left(a\right)}{\geq }\frac{H(W|Z_{1}^{N},{\tilde{Z}}_{1}^{N-1},g,\tilde{g},h_{1},\eta_{1},\eta_{2},...,\eta_{N},\eta_{e,k+1},\eta_{e,k+2},...,\eta_{e,N},{\tilde{\eta }}_{e,k+1},{\tilde{\eta }}_{e,k+2},...,{\tilde{\eta }}_{e,N-1})}{H(W)}\\ & \overset{\left(b\right)}{=}\frac{H(W|Z_{1},...,Z_{k},{\tilde{Z}}_{1},...,{\tilde{Z}}_{k},g,\tilde{g},h_{1},\eta_{1},\eta_{2},...,\eta_{N},\eta_{e,k+1},\eta_{e,k+2},...,\eta_{e,N},{\tilde{\eta }}_{e,k+1},{\tilde{\eta }}_{e,k+2},...,{\tilde{\eta }}_{e,N-1})}{H(W)}\\ & =\frac{H(W|gX_{1}+\eta_{e,1},...,gX_{k}+\eta_{e,k},\tilde{g}h_{1}X_{1}+{\tilde{\eta }}_{e,1},...,\tilde{g}h_{k}X_{k}+{\tilde{\eta }}_{e,k},g,\tilde{g},h_{1})}{H(W)}\\ & \overset{\left(c\right)}{=}\frac{H(W)-I(W|gX_{1}+\eta_{e,1},...,gX_{k}+\eta_{e,k},\tilde{g}h_{1}X_{1}+{\tilde{\eta }}_{e,1},...,\tilde{g}h_{k}X_{k}+{\tilde{\eta }}_{e,k},g,\tilde{g},h_{1})}{H(W)}\\ & =\frac{1}{H(W)}\{H(W)-[h(gX_{1}+\eta_{e,1},...,gX_{k}+\eta_{e,k},\tilde{g}h_{1}X_{1}+{\tilde{\eta }}_{e,1},...,\tilde{g}h_{k}X_{k}+{\tilde{\eta }}_{e,k},g,\tilde{g},h_{1})\\ & -h(gX_{1}+\eta_{e,1},...,gX_{k}+\eta_{e,k},\tilde{g}h_{1}X_{1}+{\tilde{\eta }}_{e,1},...,\tilde{g}h_{k}X_{k}+{\tilde{\eta }}_{e,k},g,\tilde{g},h_{1}|W)]\}\\ & \overset{\left(d\right)}{=}\frac{1}{H(W)}\{H(W)-[h(gX_{1}+\eta_{e,1},...,gX_{k}+\eta_{e,k},\tilde{g}h_{1}X_{1}+{\tilde{\eta }}_{e,1},...,\tilde{g}h_{k}X_{k}+{\tilde{\eta }}_{e,k},g,\tilde{g},h_{1})\\ & -h(\eta_{e,1},...,\eta_{e,k},{\tilde{\eta }}_{e,1},...,{\tilde{\eta }}_{e,k},g,\tilde{g},h_{1})]\}\\ & \overset{\left(e\right)}{\geq }\frac{H(W)+h(\eta_{e,1})+...+h(\eta_{e,k})+h({\tilde{\eta }}_{e,1})+...+h({\tilde{\eta }}_{e,k})+h(g,\tilde{g},h_{1})}{H(W)}\\ & -\frac{h(gX_{1}+\eta_{e,1})+...+h(gX_{k}+\eta_{e,k})+h(\tilde{g}h_{1}X_{1}+{\tilde{\eta }}_{e,1})+...+h(\tilde{g}h_{1}X_{k}+{\tilde{\eta }}_{e,k})+h(g,\tilde{g},h_{1})}{H(W)}\\ & \geq \frac{1}{H(W)}\{H(W)+h(\eta_{e,1})+...+h(\eta_{e,k})+h({\tilde{\eta }}_{e,1})+...+h({\tilde{\eta }}_{e,k})\\ & -h(gX_{1}+\eta_{e,1})-...-h(gX_{k}+\eta_{e,k})-h(\tilde{g}h_{1}X_{1}+{\tilde{\eta }}_{e,1})-...-h(\tilde{g}h_{1}X_{k}+{\tilde{\eta }}_{e,k})\}\\ & \overset{\left(f\right)}{\geq }1-\frac{k\{\log(1+\frac{P|g{|}^{2}}{\sigma_{e}^{2}})+\log(1+\frac{P|\tilde{g}h{|}^{2}}{{\tilde{\sigma }}_{e}^{2}})\}}{H(W)}\\ & \overset{\left(g\right)}{=}1-\frac{k\{\log(1+\frac{P|{g|}^{2}}{\sigma_{e}^{2}})+\log(1+\frac{P|{\tilde{g}h|}^{2}}{{\tilde{\sigma }}_{e}^{2}})\}}{N\cdot {ℛ}_{fc}^{f}\left(N,\epsilon \right)}, \end{array} \end{array} \end{array}$$ (105)

where (a) follows from H(X∣Y)≥H(X∣Y,Z), (b) follows from that $X_{i}(k+1\leq i\leq N)$ is a function of the channel noise, when the channel noise is known, *X*_*i*_, $Z_{i}=gX_{i}+\eta_{e,i}(k+1\leq i\leq N)$ and ${\tilde{Z}}_{i}=\tilde{g}(h_{1}X_{i}+\eta_{i})+{\tilde{\eta }}_{e,i}(k+1\leq i\leq N-1)$ are all known, (c) follows from H(W∣X,Y)=H(W)−I(W;X,Y), (d) follows from that $X_{1},...,X_{k}$ is a function of θ, *W* and $\left(\eta_{e,1},...,\eta_{e,k},{\tilde{\eta }}_{e,1},...,{\tilde{\eta }}_{e,k},g,\tilde{g},h_{1}\right)$ are mutually independent, (e) follows from that $h(gX_{1}+\eta_{e,1},...,gX_{k}+\eta_{e,k},\tilde{g}h_{1}X_{1}+{\tilde{\eta }}_{e,1},...,\tilde{g}h_{k}X_{k}+{\tilde{\eta }}_{e,k},g,\tilde{g},h_{1})\leq h(gX_{1}+\eta_{e,1})+...+h(gX_{k}+\eta_{e,k})+h(\tilde{g}h_{1}X_{1}+{\tilde{\eta }}_{e,1})$$+...+h(\tilde{g}h_{1}X_{k}+{\tilde{\eta }}_{e,k})+h(g,\tilde{g},h_{1})$, $\left(\eta_{e,1},...,\eta_{e,k},{\tilde{\eta }}_{e,1},...,{\tilde{\eta }}_{e,k}\right)$ and $\left(g,\tilde{g},h_{1}\right)$ are mutually independent, (f) follows from


$$\begin{array}{l} \begin{array}{l} \begin{array}{ll} & h(gX_{i}+\eta_{e,i})-h(\eta_{e,i})\leq \log\left\{\pi e[E(gX_{i}X_{i}^{*}g^{*})+\sigma_{e}^{2}]\right\}-\log(\pi e\sigma_{e}^{2})\\ & =\log\left\{\pi e(P|g{|}^{2}+\sigma_{e}^{2})\right\}-\log(\pi e\sigma_{e}^{2})=\log(1+\frac{P|g{|}^{2}}{\sigma_{e}^{2}}), \end{array} \end{array} \end{array}$$ (106)

similarly, we can get $h(\tilde{g}hX_{i}+{\tilde{\eta }}_{e,i})-h({\tilde{\eta }}_{e,i})\leq \log(1+\frac{P|\tilde{g}h{|}^{2}}{{\tilde{\sigma }}_{e}^{2}})$, (g) follows from $H(W)=N\cdot {ℛ}_{fc}^{f}(N,\epsilon )$. Therefore, the proof is completed.

### 4.2 The fading wiretap channel with intermittent noisy feedback

**Fig 15:**
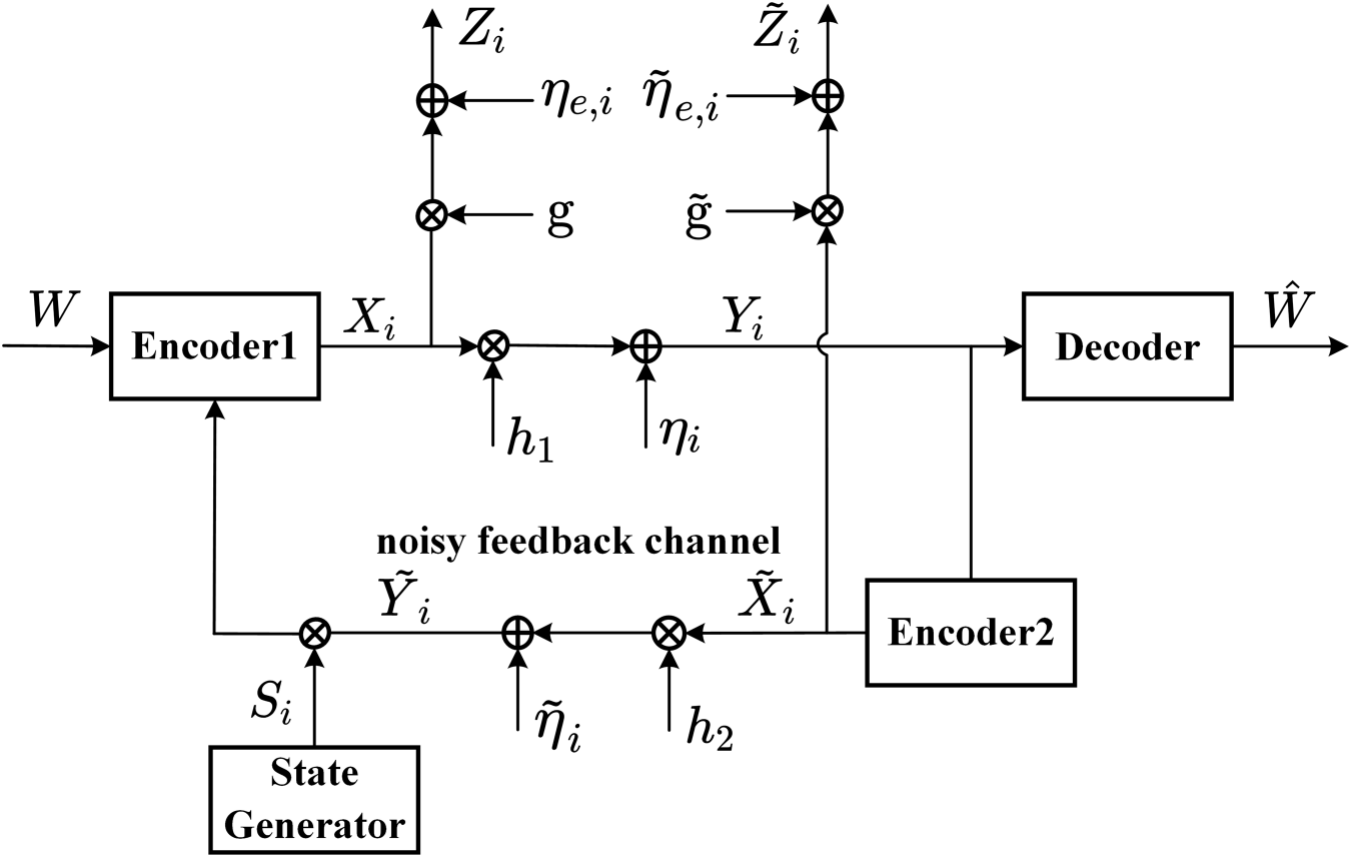
The fading wiretap channel with intermittent noisy feedback.

Fig 15 shows the fading wiretap channel with intermittent noisy feedback. In this model, the wiretap channel is given by


$$\begin{array}{l} \begin{array}{l} \begin{array}{ll} & Z_{i}=gX_{i}+\eta_{e,i},1\leq i\leq N\\ & {\tilde{Z}}_{i}=\tilde{g}{\tilde{X}}_{i}+{\tilde{\eta }}_{e,i},1\leq i\leq N-1, \end{array} \end{array} \end{array}$$ (107)

where $X_{i}\in \mathbb{C}$, ${\tilde{X}}_{i}\in \mathbb{C}$ are the input of the feedforward channel and the feedback channel at time instant *i*. The $\eta_{i}$, ${\tilde{\eta }}_{i}$, $\eta_{e,i}$ and ${\tilde{\eta }}_{e,i}$ are i.i.d as $CN(0,\sigma^{2})$, $CN(0,{\tilde{\sigma }}^{2})$, $CN(0,{\sigma_{e}}^{2})$ and $CN(0,{\tilde{\sigma_{e}}}^{2})$, respectively. $g,\tilde{g}\in \mathbb{C}$ are the fading coefficients of the eavesdropping channel.

**Definition:**

The uncertainty of the eavesdropper (also called the secrecy level) is defined as


$$\begin{array}{l} \Delta =\frac{H(W|Z_{1}^{N},{\tilde{Z}}_{1}^{N-1},h_{1},h_{2},g,\tilde{g})}{H(W)},\;0\leq \Delta \leq 1, \end{array}$$ (108)

where $Z_{1}^{N}=(Z_{1},Z_{2},...,Z_{N})$ and ${\tilde{Z}}_{1}^{N-1}=({\tilde{Z}}_{1},{\tilde{Z}}_{2},...,{\tilde{Z}}_{N-1})$, Δ=1 corresponds to perfect secrecy. We also define until the instant


$$\begin{array}{l} k(1\leq k\leq \delta_{F}(N-1)), \end{array}$$ (109)

the codeword containing the θ is successfully sent and feedback.

*Theorem 6*: For given coding blocklength *N* and decoding error probability ϵ, the lower bound on the security level of the fading channel with intermittent noisy feedback is given by


$$\begin{array}{l} \Delta_{II}\geq 1-\frac{k\log(1+\frac{P|g{|}^{2}}{\sigma_{e}^{2}})+(N-1)\log(1+\frac{\tilde{P}|\tilde{g}{|}^{2}}{{\tilde{\sigma }}_{e}^{2}})}{N\cdot {ℛ}_{fc}^{nf}\left(N,\epsilon \right)}, \end{array}$$ (110)

where $k\log(1+\frac{P|g{|}^{2}}{\sigma_{e}^{2}})$ and $\left(N-1)\log(1+\frac{\tilde{P}|\tilde{g}{|}^{2}}{{\tilde{\sigma }}_{e}^{2}}\right)$ represent the information leaked through the feedforward channel and the feedback channel, respectively.

**Proof.** From (108), the $H(W|Z_{1}^{N},{\tilde{Z}}_{1}^{N-1},h_{1},h_{2},g,\tilde{g},V_{1}^{N-1})$ is given by


$$\begin{array}{l} \begin{array}{l} \begin{array}{ll} & H(W|Z_{1}^{N},{\tilde{Z}}_{1}^{N-1},h_{1},h_{2},g,\tilde{g},V_{1}^{N-1})\\ & \overset{\left(a\right)}{\geq }H(W|Z_{1}^{N},{\tilde{Z}}_{1}^{N-1},h_{1},h_{2},g,\tilde{g},V_{1}^{N-1},\eta_{1}^{N},{\tilde{\eta }}_{1}^{N-1},\eta_{e,k+1}^{N})\\ & =H(W|\underset{Z_{1}}{\underbrace{gX_{1}+\eta_{e,1}}},\underset{Z_{2}}{\underbrace{gX_{2}+\eta_{e,2}}},...,\underset{Z_{N}}{\underbrace{gX_{N}+\eta_{e,N}}},\underset{{\tilde{Z}}_{1}}{\underbrace{\tilde{g}{\tilde{X}}_{1}+{\tilde{\eta }}_{e,1}}},\underset{{\tilde{Z}}_{2}}{\underbrace{\tilde{g}{\tilde{X}}_{2}+{\tilde{\eta }}_{e,2}}},...,\\ & \underset{{\tilde{Z}}_{N-1}}{\underbrace{\tilde{g}{\tilde{X}}_{N-1}+{\tilde{\eta }}_{e,N-1}}},h_{1},h_{2},g,\tilde{g},V_{1}^{N-1},\eta_{1}^{N},{\tilde{\eta }}_{1}^{N-1},\eta_{e,k+1}^{N})\\ & \overset{\left(b\right)}{=}H(W|gX_{1}+\eta_{e,1},...,gX_{k}+\eta_{e,k},\tilde{g}{\tilde{X}}_{1}+{\tilde{\eta }}_{e,1},\tilde{g}{\tilde{X}}_{2}+{\tilde{\eta }}_{e,2},...,\tilde{g}{\tilde{X}}_{N-1}+{\tilde{\eta }}_{e,N-1},\\ & h_{1},h_{2},g,\tilde{g},V_{1}^{N-1},\eta_{1}^{N},{\tilde{\eta }}_{1}^{N-1},\eta_{e,k+1}^{N})\\ & \overset{\left(c\right)}{\geq }H(W)+h(\eta_{e,1},...,\eta_{e,k},\eta_{e,1},{\tilde{\eta }}_{e,2},...,{\tilde{\eta }}_{e,N-1},h_{1},h_{2},g,\tilde{g},V_{1}^{N-1},\eta_{1}^{N},{\tilde{\eta }}_{1}^{N-1},\eta_{e,k+1}^{N})\\ & -[h(gX_{1}+\eta_{e,1})+...+h(gX_{k}+\eta_{e,k})+h(\tilde{g}{\tilde{X}}_{1}+{\tilde{\eta }}_{e,1})+h(\tilde{g}{\tilde{X}}_{2}+{\tilde{\eta }}_{e,2})+...+h(\tilde{g}{\tilde{X}}_{N-1}+{\tilde{\eta }}_{e,N-1})\\ & +h(h_{1},h_{2},g,\tilde{g},V_{1}^{N-1},\eta_{1}^{N},{\tilde{\eta }}_{1}^{N-1},\eta_{e,k+1}^{N})]\\ & \overset{\left(d\right)}{=}H(W)+h(\eta_{e,1})+...+h(\eta_{e,k})+h({\tilde{\eta }}_{e,1})+h({\tilde{\eta }}_{e,2})+...+h({\tilde{\eta }}_{e,N-1})-[h(gX_{1}+\eta_{e,1})\\ & +...+h(gX_{k}+\eta_{e,k})+h(\tilde{g}{\tilde{X}}_{1}+{\tilde{\eta }}_{e,1})+h(\tilde{g}{\tilde{X}}_{2}+{\tilde{\eta }}_{e,2})+...+h(\tilde{g}{\tilde{X}}_{N-1}+{\tilde{\eta }}_{e,N-1})]\\ & \overset{\left(e\right)}{\geq }H(W)-k\log(1+\frac{P|g{|}^{2}}{\sigma_{e}^{2}})-(N-1)\log(1+\frac{\tilde{P}|\tilde{g}{|}^{2}}{{\tilde{\sigma }}_{e}^{2}}), \end{array} \end{array} \end{array}$$

where (a) follows from H(X∣Y)≥H(X∣Y,Z), (b) follows from that $X_{i}(k+1\leq i\leq N)$ is a function of the channel noise, (c) follows from h(X∣Y,Z)=h(X,Y,Z)−h(Y,Z)≥h(X,Y,Z)−h(Y)−h(Z), *W* and $\left(\eta_{e,1}...,\eta_{e,k},{\tilde{\eta }}_{e,1},{\tilde{\eta }}_{e,2},...,{\tilde{\eta }}_{e,N-1},h_{1},h_{2},g,\tilde{g},V_{1}^{N-1},\eta_{1}^{N},{\tilde{\eta }}_{1}^{N-1},\eta_{e,k+1}^{N}\right)$ are mutually independent, (d) follows from that the elements in $(\eta_{e,1}...,\eta_{e,k},{\tilde{\eta }}_{e,1},{\tilde{\eta }}_{e,2},...,{\tilde{\eta }}_{e,N-1},$ $h_{1},h_{2},g,\tilde{g},V_{1}^{N-1},\eta_{1}^{N},{\tilde{\eta }}_{1}^{N-1},\eta_{e,k+1}^{N})$ are mutually independent, (e) follows from that


$$\begin{array}{l} \begin{array}{l} \begin{array}{ll} h(gX_{i}+\eta_{e,i})-h(\eta_{e,i}) & \leq \log(\pi eE[(gX_{i}+\eta_{e,i})(gX_{i}+\eta_{e,i}{)}^{*}])-\log(\pi e\sigma_{e}^{2})\\ & =\log(\pi e(P|g{|}^{2}+\sigma_{e}^{2}))-\log(\pi e\sigma_{e}^{2})\\ & =\log(1+\frac{P|g{|}^{2}}{\sigma_{e}^{2}}), \end{array} \end{array} \end{array}$$ (111)

similarly, we can get $h(\tilde{g}{\tilde{X}}_{i}+{\tilde{\eta }}_{e,i})-h({\tilde{\eta }}_{e,i})\leq \log(1+\frac{\tilde{P}|\tilde{g}{|}^{2}}{{\tilde{\sigma }}_{e}^{2}})$. Substituting (111) into (108), we can obtain


$$\begin{array}{l} \begin{array}{l} \begin{array}{ll} \Delta_{II} & \geq \frac{1}{H(W)}\left\{H(W)-k\log(1+\frac{P|g{|}^{2}}{\sigma_{e}^{2}})-(N-1)\log(1+\frac{\tilde{P}|\tilde{g}{|}^{2}}{{\tilde{\sigma }}_{e}^{2}})\right\}\\ & =1-\frac{k\log(1+\frac{P|g{|}^{2}}{\sigma_{e}^{2}})+(N-1)\log(1+\frac{\tilde{P}|\tilde{g}{|}^{2}}{{\tilde{\sigma }}_{e}^{2}})}{N\cdot {ℛ}_{fc}^{nf}\left(N,\epsilon \right)}, \end{array} \end{array} \end{array}$$ (112)

therefore, the proof is completed.

### 4.3 Numerical examples

**Fig 16:**
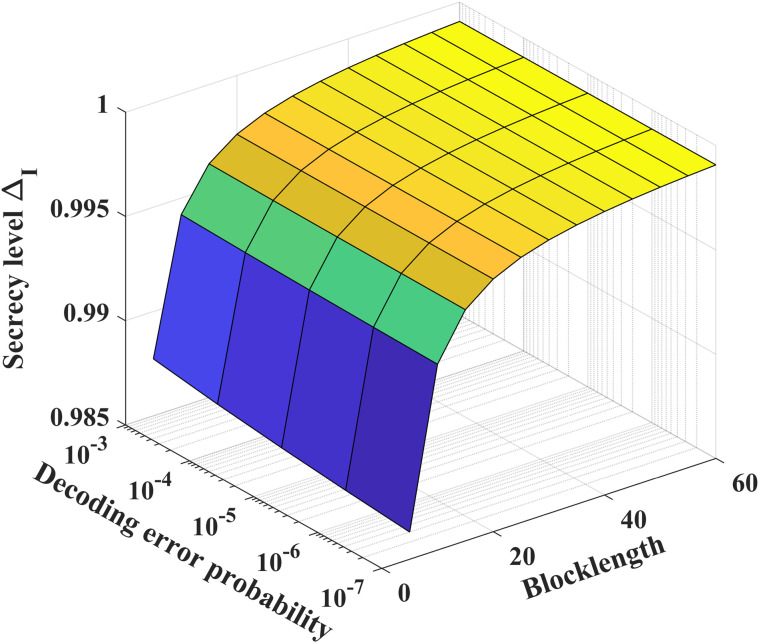
The relationship between $\Delta_{I}$, *P*_*e*_ and *N* for *P* = 10 dB, $\delta_{F}=0.2$, $h_{1},g,\tilde{g}\sim CN(0,1)$, *k* = 1, $\sigma_{e}^{2}=100$, ${\tilde{\sigma }}_{e}^{2}=1000$ and $\sigma^{2}=1$.

**Fig 17:**
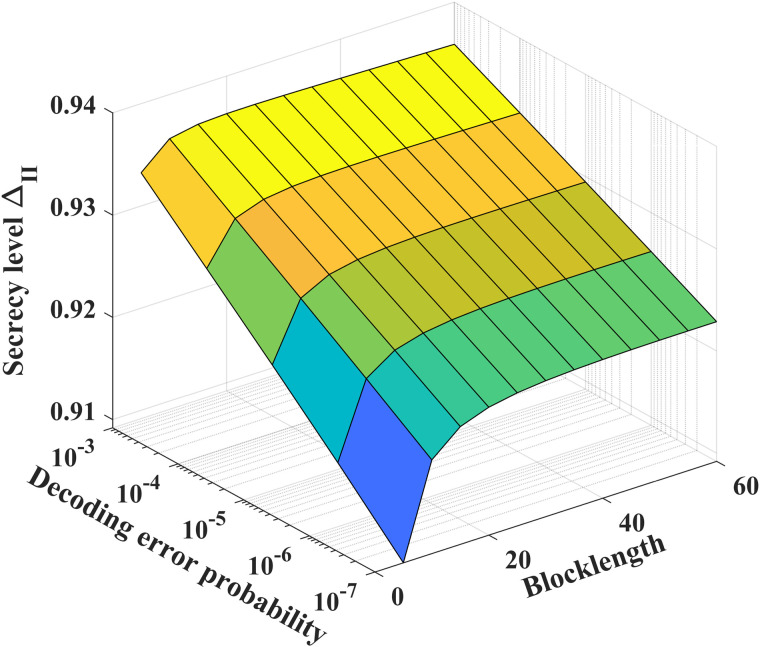
The relationship between$\Delta_{II}$, *P*_*e*_ and *N* for *P* = 10 dB, $\tilde{P}=20$ dB, $\delta_{F}=0.2$, $h_{1},h_{2},g,\tilde{g}\sim CN(0,1)$, $\sigma_{e}^{2}=100$, ${\tilde{\sigma }}_{e}^{2}=1000$, *k* = 1 and ${\tilde{\sigma }}^{2}=\sigma^{2}=1$.

Fig 16 and 17 show the relationship between secrecy level, decoding error probability and coding blocklength. As the blocklength *N* and the decoding error probability *P*_*e*_ increase, the security level $\Delta_{I}$ and $\Delta_{II}$ gradually rises. To achieve a desired decoding error probability (10^−3^) and the coding blocklength for the noiseless/noisy feedback case is about 60, $\Delta_{I}$ can achieve perfect security, but $\Delta_{II}$ can not guarantee perfect security.

**Fig 18:**
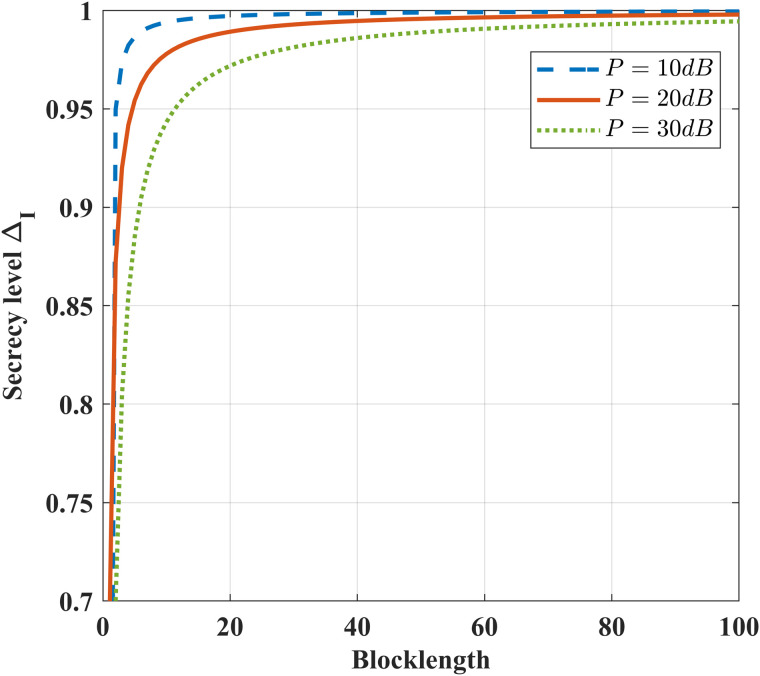
The impact of *N* on security level $\Delta_{I}$ under different *P* and $\epsilon =10^{-6}$, *k* = 1,$h_{1},g,\tilde{g}\sim CN(0,1)$, $\delta_{F}=0.2$, $\sigma^{2}=1$, $\sigma_{e}^{2}=100$ and${\tilde{\sigma }}_{e}^{2}=1000$.

Fig 18 illustrates the impact of the coding blocklength on security level $\Delta_{I}$ under different power *P*. As the blocklength increases, $\Delta_{I}$ gradually improves and approaches perfect security. This is because, in the proposed scheme with k = 1, information leakage only occurs at initial transmission instant. With increasing blocklength, the average information leakage of the scheme asymptotically approaches zero. For given blocklength, the higher transmit power *P* leads to lower security level.

**Fig 19:**
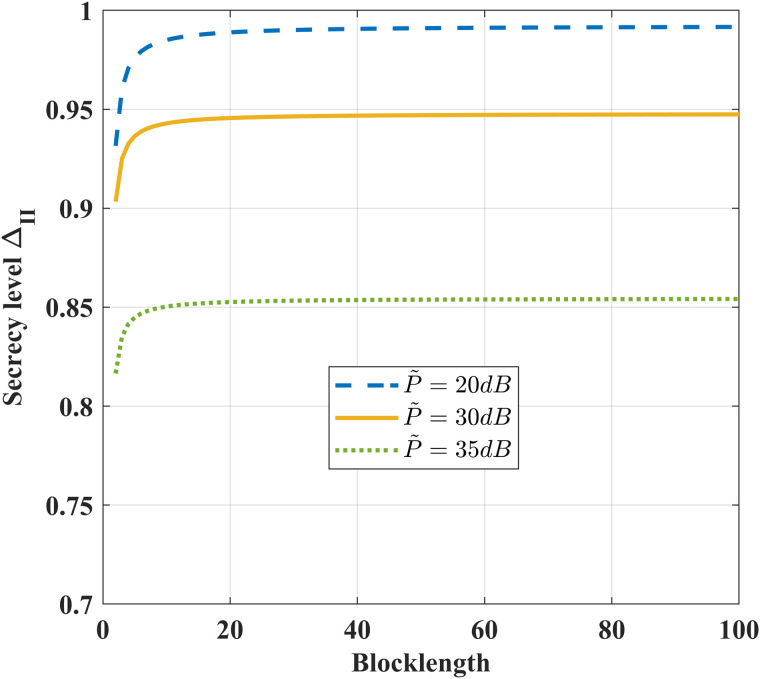
The impact of *N* on security level $\Delta_{I}$ under different *P* and $\epsilon =10^{-6}$, *k* = 1, $h_{1},g,\tilde{g}\sim CN(0,1)$, $\delta_{F}=0.2$, $\sigma^{2}=1$, $\sigma_{e}^{2}=100$ and ${\tilde{\sigma }}_{e}^{2}=1000$.

Fig 19 illustrates the impact of the coding blocklength on security level $\Delta_{II}$ under different feedback power $\tilde{P}$. As the blocklength increases, $\Delta_{II}$ gradually improves. For given blocklength, as the feedback power $\tilde{P}$ increases, the security level $\Delta_{II}$ correspondingly decreases. This occurs because the feedback information at each transmission instant is intercepted by the eavesdropper, and higher feedback power directly improves the eavesdropper’s reception quality, thereby increasing information leakage. Achieving perfect security requires the condition that ${\tilde{\sigma }}_{e}^{2}\gg \tilde{P}$ and sufficiently large blocklength.

**Fig 20:**
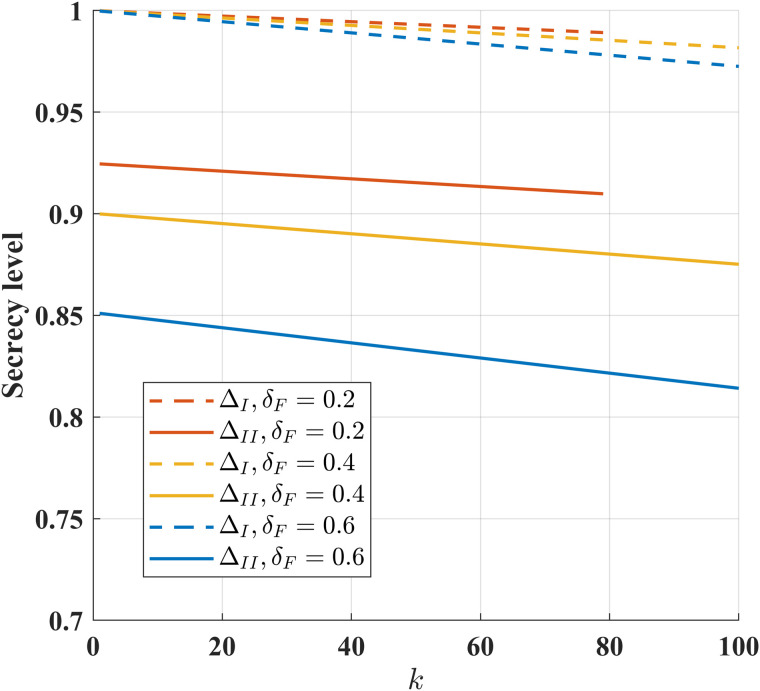
The impact of instant *k* on security levels $\Delta_{I}$ and $\Delta_{II}$ under different feedback failure probabilities and *P* = 10 dB, $\tilde{P}=20$ dB, $\epsilon =10^{-6}$, $h_{1},h_{2},g,\tilde{g}\sim CN(0,1)$, *N* = 400, ${\tilde{\sigma }}^{2}=\sigma^{2}=1$, $\sigma_{e}^{2}=100$ and ${\tilde{\sigma }}_{e}^{2}=1000$.

As shown in Fig 20, we analyze how the instant *k* affects both $\Delta_{I}$ and $\Delta_{II}$ under different feedback failure probability $\delta_{F}$. Under a fixed $\delta_{F}$, $\Delta_{I}$ and $\Delta_{II}$ decrease monotonically with increasing of instant *k*. For given instant *k*, higher $\delta_{F}$ leads to degraded security levels $\Delta_{I}$ and $\Delta_{II}$. These results indicate that ensuring system security requires minimizing both the $\delta_{F}$ and the instant *k*.

## 5 Conclusion and future work

In this paper, we propose SK-type highly efficient coding schemes for channels with intermittent feedback, in both noise-free and noisy feedback cases. Numerical results show that to achieve a desired decoding error probability, the coding blocklength of our scheme is significantly shorter, and the encoding-decoding complexity is in linear proportion to the coding blocklength. Besides this, it was shown that when the coding blocklength is not long enough, our scheme almost approaches the PLS requirement, namely, weak secrecy. Though we have shown that our proposed schemes outperform existing ones in the literature from several aspects, some practical implementation challenges still exist, and they are given below.

- *Synchronization*: Following the frame synchronization schemes [28] for channels with feedback, we note that a short synchronization phase can be embedded at the beginning of each transmission frame to align the transmitter and receiver with respect to the effective operational state of the feedback-assisted SK-type scheme. Due to the state-forgetting property, the impact of initial synchronization mismatch becomes negligible after a finite transient period and may not affect the performance of following message transmissions.
- *Dither Generation*: The dither sequence is generated using a shared pseudo random seed, which can be established or refreshed during the synchronization phase or via feedback signaling. Occasional loss of dither alignment only affects a finite number of symbols and may not alter the asymptotic rate, reliability, or secrecy guarantees.
- *Mitigation strategy for shared randomness assumptions*: If no randomness is shared by the receiver and transmitter, one possible mitigation strategy is given below. At the first time instant, the transmitter sends $X_{1}=\sqrt{12P}\theta$, the receiver estimates θ by ${\hat{\theta }}_{1}=\frac{Y_{1}}{\sqrt{12P}}=\theta +\frac{\eta_{1}}{\sqrt{12P}}$, and he feeds back ${\tilde{X}}_{1}=\sqrt{\frac{\tilde{P}}{Var(Y_{1})}}Y_{1}$ to the transmitter. At time instant i(2≤i≤N), the transmitter obtains a noisy version of the previous feedforward channel output *Y*_*i*−1_, which is given by ${\hat{Y}}_{i-1}=\frac{{\tilde{Y}}_{i-1}}{\sqrt{\frac{\tilde{P}}{Var(Y_{i-1})}}}=Y_{i-1}+\frac{{\tilde{\eta }}_{i-1}}{\sqrt{\frac{\tilde{P}}{Var(Y_{i-1})}}}$. Hence, by ${\hat{Y}}_{i-1}$, the transmitter can calculate a noisy version of the receiver’s estimation error, and forwards it for helping the receiver update his estimation of θ, namely, the transmitter’s codeword at time instant *i* is given by
  $$\begin{array}{ll} {\tilde{\varepsilon }}_{i-1} & =\beta_{i-1}{\hat{Y}}_{i-1}-X_{i-1}\\ & =\underset{\text{Receiver's estimation of }X_{i-1}}{\beta_{i-1}Y_{i-1}}-X_{i-1}+\frac{\beta_{i-1}{\tilde{\eta }}_{i-1}}{\sqrt{\frac{\tilde{P}}{Var(Y_{i-1})}}}. \end{array}$$ (113)

From (113), it is easy to see that if setting $\beta_{1}=1$, then ${\tilde{\varepsilon }}_{1}=\eta_{1}+\frac{{\tilde{\eta }}_{1}}{\sqrt{\frac{\tilde{P}}{Var(Y_{1})}}}$, and ${\tilde{\varepsilon }}_{i}=f(\eta^{i},{\tilde{\eta }}^{i})$ which indicates that the codewords from time instant 2 to *N* are combinations of channel noises. Then along the lines of proof in [13], it is easy to prove that this strategy achieves perfect secrecy. However, this strategy may lead to significant degradation in transmission performance since it involves additional channel noise $\frac{\beta_{i-1}{\tilde{\eta }}_{i-1}}{\sqrt{\frac{\tilde{P}}{Var(Y_{i-1})}}}$ in the feedforward transmission at each time instant.

- *Imperfect CSI at the transceiver*: If the transmitter does not know the perfect CSI, it will cause additional encoding-decoding error in the coding performance, and this kind of error will be accumulated, resulting in the decoding failure since the receiver’s decoding error probability does not vanish as coding blocklength tends to infinity.

Possible future work includes:

- Extension of our SK-type schemes to multi-user channel models is challenging, e.g., for the two-user Gaussian multiple-access channel with intermittent feedback, the SK-type scheme does not work since the two feedback channels may not be in feedback success at the same time, while this synchronization is the key to the success of original SK-type scheme used in this model.
- Here note that as shown in [20], the performance of the SK-type scheme for the noisy feedback case relies heavily on the sufficiently large feedback power. However, in covert communication [29,30], the encoding power should be sufficiently small, and this leads to the extension of our schemes to covert communication scenario becomes challenging.
- For the channels with feedback delay, how to design SK-type scheme is also interesting and challenging. Specifically, if the feedback delay is a constant, namely, at all time instants, the delay time is fixed. It is easy to extend to our schemes to such a case, for example, if the delay time is *d* (*d* ≥ 2, here note that *d* = 1 is exactly the same as the model of this paper), From time 1 to *d* − 1, the transmitter sends nothing, and at time *d*, he sends the initial message as SK scheme does, and from *d* + 1 to *N*, the iteration is the same as those given in this paper. Along the lines of performance analysis in this paper, it is not difficult to show that the asymptotic rate is the same as that of this paper, while the finite blocklength rate is related with *d*. On the other hand, if the feedback delay changes with time, for example, the feedback delay *d* is a random variable, which takes values in a finite set $\left\{1,2,...,d_{max}\right\}$, a trivial feedback coding scheme for such case is described as follows: Dividing the *N* coding blocklength into *L* blocks with each block consisting of *d*_*max*_ time instants, then using the first block to transmit the initial message and the following *L* − 1 blocks to transmit the receiver’s estimation errors as the SK scheme does, a feedback coding scheme for the variable feedback delay case is obtained. It is obvious that such scheme suffers from performance degradation as the number of iteration decreases in fact, and it is challenging to design superior feedback coding schemes for this case, which will be our future work.

## Appendix

### A Error probability analysis in the proof of theorem 2

The decoding error probability *P*_*e*_ of *W* is bounded as follows. Recall that the transmitter computes ${\tilde{\varepsilon }}_{a_{i-1}}$, via a modulo operation (34). For any time instant *i* (2 ≤ *i* ≤ *N*) and *S*_*i*−1_ = 1, define $E_{a_{i-1}}$ as a modulo-aliasing error occurs during transmission, i.e.,


$$E_{a_{i-1}}\overset{\text{def}}{=}\{\gamma_{i-1}\varepsilon_{a_{i-1}}+{\tilde{\eta }}_{i-1}\notin [-\frac{d}{2},\frac{d}{2})\}.$$ (114)

Furthermore, the final estimation of θ is ${\hat{\theta }}_{a_{N}}=\theta +\varepsilon_{a_{N}}$, define $E_{a_{N}}$ to be the decoding error event at time instant *N*, i.e.,


$$E_{a_{N}}≜\{\varepsilon_{a_{N}}\notin [-\frac{1}{2\cdot 2^{N{ℛ}^{nf}}},\frac{1}{2\cdot 2^{N{ℛ}^{nf}}})\}.$$ (115)

Then the decoding error probability is upper bounded by


$$\begin{array}{ll} P_{e} & ⩽\mathrm{Pr}\left(\overset{a_{N-1}}{\underset{k=1}{⋃}}E_{k}\cup E_{a_{N}}\right)\\ & =\mathrm{Pr}\left(\overset{a_{N-1}}{\underset{k=1}{⋃}}E_{k}\right)+\mathrm{Pr}\left(\overset{a_{N-1}}{\underset{k=1}{⋂}}E_{k}^{c}\cap E_{a_{N}}\right)\\ & =\sum_{k=1}^{a_{N-1}}\mathrm{Pr}(E_{k}^{{\;}^{\prime }})+\mathrm{Pr}(E_{a_{N}}^{{\;}^{\prime }}), \end{array}$$ (116)

where $E_{k}^{{\;}^{\prime }}≜{⋂}_{j=1}^{k-1}E_{{\;}_{j}}^{c}\cap E_{k}$, $E_{a_{N}}^{{\;}^{\prime }}≜{⋂}_{k=1}^{a_{N-1}}E_{k}^{c}\cap E_{a_{N}}$ and *E*^*c*^ is the complement of the event *E*. $\mathrm{Pr}(E_{a_{i-1}}^{{\;}^{\prime }})$ (*i*={2,...,*N*}) is denoted as the probability that modulo-aliasing error occurs and there is no such error occurs during all previous times, and $\mathrm{Pr}(E_{a_{N}}^{{\;}^{\prime }})$ indicates the error probability of the final decoding without any modulo-aliasing error occurring before.

As mentioned before, the target error probability is set to ϵ, then let $\mathrm{Pr}(E_{a_{N}}^{{\;}^{\prime }})=\frac{\epsilon }{2}$, we have


$$\mathrm{Pr}(E_{a_{i-1}}^{{\;}^{\prime }})=\frac{\epsilon }{2a_{N-1}}.$$ (117)

Recalling the definition of the event $E_{a_{i-1}}^{{\;}^{\prime }}$ in (114) and $d=\sqrt{12\tilde{P}}$, since $\gamma_{i-1}\varepsilon_{a_{i-1}}+{\tilde{\eta }}_{i-1}$ is Gaussian and $\gamma_{i-1}\varepsilon_{a_{i-1}}+{\tilde{\eta }}_{i-1}\sim 𝒩(0,\gamma_{i-1}^{2}\alpha_{a_{i-1}}+{\tilde{\sigma }}^{2})$, so we obtain that


$$\begin{array}{ll} & \mathrm{Pr}(E_{a_{i-1}}^{{\;}^{\prime }})=\mathrm{Pr}\left\{\gamma_{i-1}\varepsilon_{a_{i-1}}+{\tilde{\eta }}_{i-1}\notin [-\frac{d}{2},\frac{d}{2})\right\}\\ & =2Q\left(\sqrt{\frac{3\tilde{P}}{\gamma_{i-1}^{2}\alpha_{a_{i-1}}+{\tilde{\sigma }}^{2}}}\right), \end{array}$$ (118)

to simplify the calculation, parameter *L* is introduced, which is denoted as


$$L=\frac{\tilde{P}}{\gamma_{i-1}^{2}\alpha_{a_{i-1}}+{\tilde{\sigma }}^{2}},$$ (119)

using the definition of *L*, we can obtain that


$$\gamma_{i-1}^{2}\alpha_{a_{i-1}}+{\tilde{\sigma }}^{2}=\frac{\tilde{P}}{L},$$ (120)

from (120), the modulation coefficient $\gamma_{i-1}$ of the transmitter is given by


$$\gamma_{i-1}=\sqrt{\frac{1}{\alpha_{a_{i-1}}}(\frac{\tilde{P}}{L}-{\tilde{\sigma }}^{2})}.$$ (121)

Also note that, combining the (117), (118) and (120), we can obtain the following setting of *L*


$$L=\frac{1}{3}{\left[Q^{-1}\left(\frac{\epsilon }{4a_{N-1}}\right)\right]}^{2}.$$ (122)

Since *X*_*R*,*i*_ should obey the power constraint


$$E(X_{R,i}^{2})=P_{R}$$

, we can easily obtain $\lambda_{R,i-1}$


$$\begin{array}{ll} P_{R} & =E{\left(X_{{\;}_{R,i}}\right)}^{2}=E{\left(\lambda_{{\;}_{R,i-1}}\gamma_{{\;}_{R,i-1}}{\tilde{\varepsilon }}_{{\;}_{R,a_{i-1}}}\right)}^{2}\\ & =\lambda_{R,i-1}^{2}E{\left(\gamma_{R,i-1}{\tilde{\varepsilon }}_{R,a_{i-1}}\right)}^{2}\\ & =\lambda_{R,i-1}^{2}E{\left(\gamma_{R,i-1}\varepsilon_{R,a_{i-1}}+{\tilde{\eta }}_{R,i-1}^{{\;}^{\prime }}\right)}^{2}, \end{array}$$ (123)

from (120) and (B10) it follows that


$$\lambda_{i-1}=\sqrt{L\frac{P}{\tilde{P}}}.$$ (124)

Moreover, i^*^ is defined, where $S_{i^{*}-1}=1$ and $a_{i^{*}-1}=a_{i-1}-1$, substituting $X_{i-1}=\lambda_{i^{*}-1}\gamma_{i^{*}-1}{\tilde{\varepsilon }}_{a_{i-2}}$, (121) and (124) into (37), since $\eta_{i-1}\sim 𝒩(0,\sigma^{2})$ is independent of $\varepsilon_{a_{i-2}}$ and *X*_*i*−1_, we obtained that


$$\begin{array}{ll} \beta_{i-1} & =\frac{\lambda_{i^{*}-1}\gamma_{i^{*}-1}E[\varepsilon_{a_{i-2}}^{2}]}{E[X_{i-1}^{2}]+E[\eta_{i-1}^{2}]}=\frac{\lambda_{i^{*}-1}\gamma_{i^{*}-1}\alpha_{a_{i-2}}}{P+\sigma^{2}}\\ & =\frac{\sqrt{\alpha_{a_{i-2}}SNR\cdot (1-L\cdot {\tilde{SNR}}^{-1})}}{\sigma (1+SNR)}. \end{array}$$ (125)

According to (37) and (38), we conclude that for any time instant *i* − 1 (2 ≤ *i* ≤ *N*)


$$\begin{array}{ll} \alpha_{a_{i-1}} & =E(\varepsilon_{a_{i-1}}{)}^{2}=E(\varepsilon_{a_{i-2}}-\beta_{i-1}Y_{i-1}{)}^{2}\\ & =E(\varepsilon_{a_{i-2}}{)}^{2}-\frac{E^{2}(Y_{i-1}\varepsilon_{a_{i-2}})}{E(Y_{i-1}{)}^{2}}, \end{array}$$ (126)

substituting (121), (124) and (125) into (126), we obtain


$$\alpha_{a_{i-1}}=\frac{SNR^{-1}}{12}{\left(1+\frac{SNR}{\Psi_{1}\Psi_{2}}\right)}^{1-a_{i-1}},$$ (127)

where $\Psi_{1}=1+L\cdot \frac{SNR}{\tilde{SNR}}$, $\Psi_{2}=\frac{1}{1-L\cdot {\tilde{SNR}}^{-1}}$. We also obtain $\alpha_{a_{N}}=\frac{SNR^{-1}}{12}{\left(1+\frac{SNR}{\Psi_{1}\Psi_{2}}\right)}^{-a_{N-1}}$, which will be used in the decoding error probability analysis of receiver.

According to (115), we conclude that


$$\begin{array}{ll} \mathrm{Pr}(E_{a_{N}}^{{\;}^{\prime }}) & =\mathrm{Pr}\left\{\varepsilon_{a_{N}}\notin [-\frac{1}{2\cdot 2^{N{ℛ}^{nf}}},\frac{1}{2\cdot 2^{N{ℛ}^{nf}}})\right\}\\ & =2Q\left(\frac{1}{2\cdot 2^{N{ℛ}^{nf}}}\cdot \frac{1}{\sqrt{\alpha_{a_{N}}}}\right), \end{array}$$ (128)

and


$$Q\left(\frac{1}{2\cdot 2^{N{ℛ}^{nf}}}\cdot \frac{1}{\sqrt{\alpha_{a_{N}}}}\right)=\frac{\epsilon }{4},$$ (129)

substituting $\alpha_{a_{N}}$ into (129), the achievable rate of the channel with intermittent noisy feedback is calculated by


$$\begin{array}{ll} & {ℛ}^{nf}=\frac{1}{2N}\log\left(\frac{3\cdot SNR}{[Q^{-1}(\frac{\epsilon }{4}){]}^{2}}\cdot {\left(1+\frac{SNR}{\Psi_{1}\Psi_{2}}\right)}^{a_{N-1}}\right), \end{array}$$ (130)

which completes the proof.

### B Error probability analysis in the proof of Theorem 4

Since *W* is divided into two independent uniformly distributed sub-messages (*W*_*R*_, *W*_*I*_), we define the decoding error probability of (*W*_*R*_, *W*_*I*_) as *P*_*e*,*R*_ and *P*_*e*,*I*_. The decoding error probability analysis process is the same for the real part and the imaginary part, and the corresponding decoding error probability of the real part is analyzed below. Recall that the transmitter computes ${\tilde{\varepsilon }}_{R,a_{i-1}}$, via a modulo operation (88). For any time instant *i* (2 ≤ *i* ≤ *N*) and *S*_*i*−1_ = 1, define $E_{R,a_{i-1}}$ as a modulo-aliasing error occurs during transmission, i.e.,


$$E_{R,a_{i-1}}=\left\{\gamma_{R,i-1}\varepsilon_{R,a_{i-1}}+{\tilde{\eta }}_{R,i-1}^{\prime }\notin \left[-\frac{d_{R}}{2},\frac{d_{R}}{2}\right]\right\}.$$ (B1)

Furthermore, the final estimation of θ is ${\hat{\theta }}_{R,a_{N}}=\theta_{R}+\varepsilon_{R,a_{N}}$, define $E_{R,a_{N}}$ to be the decoding error event at time instant *N*, i.e.,


$$E_{R,a_{N}}=\{\varepsilon_{R,a_{N}}\notin [-\frac{1}{2\cdot 2^{N{ℛ}_{R,fc}^{nf}}},\frac{1}{2\cdot 2^{N{ℛ}_{R,fc}^{nf}}})\}.$$ (B2)

Then the decoding error probability is upper bounded by


$$\begin{array}{ll} P_{e,R} & \leq \mathrm{Pr}\left(\overset{a_{N-1}}{\underset{k=1}{⋃}}E_{R,k}\cup E_{R,a_{N}}\right)\\ & =\mathrm{Pr}\left(\overset{a_{N-1}}{\underset{k=1}{⋃}}E_{R,k}\right)+\mathrm{Pr}\left(\overset{a_{N-1}}{\underset{k=1}{⋂}}E_{R,k}^{c}\cap E_{R,a_{N}}\right)\\ & =\sum_{k=1}^{a_{N-1}}\mathrm{Pr}(E_{R,k}^{\prime })+\mathrm{Pr}(E_{R,a_{N}}^{\prime }), \end{array}$$ (B3)

where $\mathrm{Pr}(E_{R,a_{i-1}}^{{\;}^{\prime }})$ (*i*={2,...,*N*}) is denoted as the probability that modulo-aliasing error occurs and there is no such error occurs during all previous times, and $\mathrm{Pr}(E_{R,a_{N}}^{{\;}^{\prime }})$ indicates the error probability of the final decoding without any modulo-aliasing error occurring before.

As mentioned before, the target error probability is set to ϵ, then let $\mathrm{Pr}(E_{R,a_{N}}^{{\;}^{\prime }})=\frac{\epsilon }{4}$, we have


$$\mathrm{Pr}(E_{R,a_{i-1}}^{{\;}^{\prime }})=\frac{\epsilon }{4a_{N-1}}=p_{m}.$$ (B4)

Recalling the definition of the event $E_{R,a_{i-1}}^{{\;}^{\prime }}$ in (B1) and $d_{R}=\sqrt{12{\tilde{P}}_{R}}$, since $\gamma_{R,i-1}\varepsilon_{R,a_{i-1}}+{\tilde{\eta }}_{R,i-1}$ is Gaussian, we obtain that


$$p_{m}=\mathrm{Pr}\left\{\gamma_{R,i-1}\varepsilon_{R,a_{i-1}}+{\tilde{\eta }}_{R,i-1}^{\prime }\notin \left[-\frac{d_{R}}{2},\frac{d_{R}}{2}\right]\right\}\;=2Q\left(\sqrt{\frac{3{\tilde{P}}_{R}}{E{\left(\gamma_{R,i-1}\varepsilon_{R,a_{i-1}}+{\tilde{\eta }}_{R,i-1}^{\prime }\right)}^{2}}}\right),$$ (B5)

to simplify the calculation, parameter *L* is introduced, which is denoted as


$$L=\frac{{\tilde{P}}_{R}}{\gamma_{R,i-1}^{2}\alpha_{R,a_{i-1}}+\frac{{\tilde{\sigma }}^{2}}{2\left(h_{R,2}^{2}+h_{I,2}^{2}\right)}},$$ (B6)

using the definition of *L*, we can obtain that


$$\gamma_{R,i-1}^{2}\alpha_{R,a_{i-1}}+\frac{{\tilde{\sigma }}^{2}}{2\left(h_{R,2}^{2}+h_{I,2}^{2}\right)}=\frac{{\tilde{P}}_{R}}{L}=\frac{\tilde{P}}{2L},$$ (B7)

from (B7), the modulation coefficient $\gamma_{R,i-1}$ of the transmitter is given by


$$\gamma_{R,i-1}=\sqrt{\frac{1}{\alpha_{R,a_{i-1}}}\left(\frac{\tilde{P}}{2L}-\frac{{\tilde{\sigma }}^{2}}{2\left(h_{R,2}^{2}+h_{I,2}^{2}\right)}\right)}.$$ (B8)

Also note that, from (B5) and (B7), we can obtain the following setting of *L*


$$L=\frac{1}{3}{\left[Q^{-1}(\frac{p_{m}}{2})\right]}^{2}.$$ (B9)

Since *X*_*R*,*i*_ should obey the power constraint of the transmitter from (47), we can easily obtain $\lambda_{i-1}$


$$\begin{array}{ll} P & =E(X_{i}{)}^{2}=E(\lambda_{i-1}\gamma_{i-1}{\tilde{\varepsilon }}_{a_{i-1}}{)}^{2}\\ & =\lambda_{i-1}^{2}(\gamma_{i-1}^{2}\alpha_{a_{i-1}}+{\tilde{\sigma }}^{2}), \end{array}$$ (B10)

from (B7) and (B10) it follows that


$$\lambda_{R,i-1}=\sqrt{L\frac{P}{\tilde{P}}}.$$ (B11)

Moreover, i^*^ is defined, where $S_{i^{*}-1}=1$ and $a_{i^{*}-1}=a_{i-1}-1$, substituting $X_{R,i-1}=\lambda_{R,i^{*}-1}\gamma_{R,i^{*}-1}{\tilde{\varepsilon }}_{R,a_{i-2}}$, (B8) and (B11) into (92), we obtained that


$$\begin{array}{ll} \beta_{{\;}_{R,i-1}} & =\frac{E\left(Y_{R,i-1}^{{\;}^{\prime }}\varepsilon_{R,a_{i-2}}\right)}{E{\left(Y_{R,i-1}^{{\;}^{\prime }}\right)}^{2}}=\frac{\lambda_{R,a_{i^{*}-1}}\gamma_{R,a_{i^{*}-1}}\alpha_{R,a_{i-2}}}{\frac{P}{2}+\frac{\sigma^{2}}{2\left(h_{R,1}^{2}+h_{I,1}^{2}\right)}}\\ & =\frac{\sqrt{2\alpha_{R,a_{I-2}}}}{\sigma }\cdot \frac{\sqrt{SNR\left(1-L\cdot S\tilde{N}R^{-1}{\left(|h_{2}{|}^{2}\right)}^{-1}\right)}}{SNR+{\left(h_{1}{|}^{2}\right)}^{-1}}. \end{array}$$ (B12)

Substituting (B8), (B11) and (B12) into $\varepsilon_{R,a_{i-1}}=\varepsilon_{R,a_{i-2}}-\beta_{R,i-1}Y_{R,i-1}^{\prime }$, we conclude that for any time instant *i* − 1 (2 ≤ *i* ≤ *N*)


$$\begin{array}{ll} \alpha_{R,a_{i-1}} & =E{\left(\varepsilon_{R,a_{i-1}}\right)}^{2}=E{\left(\varepsilon_{R,a_{i-2}}\right)}^{2}-\frac{E^{2}\left(Y_{R,i-1}^{\prime }\varepsilon_{R,a_{i-2}}\right)}{E{\left(Y_{R,i-1}^{\prime }\right)}^{2}}\\ & =SNR^{-1}\cdot {\left(|h_{1}{|}^{2}\right)}^{-1}{\left(1+\frac{SNR\cdot \left(|h_{1}{|}^{2}\right)}{\psi_{1}\psi_{2}}\right)}^{1-a_{i-1}}, \end{array}$$ (B13)

where


$$\begin{array}{ll} & \psi_{1}=1+L\cdot SNR\cdot S\tilde{N}R^{-1}\cdot \left(|h_{1}{|}^{2}\right){\left(|h_{2}{|}^{2}\right)}^{-1}\\ & \psi_{2}={\left(1-L\cdot S\tilde{N}R^{-1}{\left(|h_{2}{|}^{2}\right)}^{-1}\right)}^{-1}, \end{array}$$ (B14)

According to (B2), we conclude that


$$\begin{array}{ll} \mathrm{Pr}(E_{R,a_{N}}^{{\;}^{\prime }}) & =\mathrm{Pr}\left\{\varepsilon_{R,a_{N}}\notin \left[-\frac{\sqrt{3}}{2^{NR_{R,fc}^{nf}}},\frac{\sqrt{3}}{2^{NR_{R,fc}^{nf}}}\right]\right\}\\ & =2Q\left(\sqrt{\frac{3}{\alpha_{R,a_{N}}\cdot 2^{2NR_{R,fc}^{nf}}}}\right), \end{array}$$ (B15)

and


$$2Q\left(\sqrt{\frac{3}{\alpha_{R,a_{N}}\cdot 2^{2NR_{R,fc}^{nf}}}}\right)=\frac{\epsilon }{4},$$ (B16)

substituting (B13) into (B16), the achievable rate ${ℛ}_{R,fc}^{nf}$ is calculated by


$${ℛ}_{R,fc}^{nf}=\frac{1}{2N}log\left(\frac{3}{{\left[Q^{-1}\left(\frac{\epsilon }{8}\right)\right]}^{2}}\cdot SNR\cdot \left(|h_{1}{|}^{2}\right){\left(1+\frac{SNR\cdot \left(|h_{1}{|}^{2}\right)}{\psi_{1}\psi_{2}}\right)}^{a_{N-1}}\right),$$ (B17)

similarly, we can get ${ℛ}_{I,fc}^{nf}$


$${ℛ}_{I,fc}^{nf}=\frac{1}{2N}log\left(\frac{3}{{\left[Q^{-1}\left(\frac{\epsilon }{8}\right)\right]}^{2}}\cdot SNR\cdot \left(|h_{1}{|}^{2}\right){\left(1+\frac{SNR\cdot \left(|h_{1}{|}^{2}\right)}{\psi_{1}\psi_{2}}\right)}^{a_{N-1}}\right),$$ (B18)

From (B17) and (B18), the achievable rate of the fading channel with intermittent noisy feedback is calculated by


$$\begin{array}{ll} {ℛ}_{fc}^{nf} & ={ℛ}_{R,fc}^{nf}+{ℛ}_{I,fc}^{nf}\\ & =\frac{1}{N}\log\left(\frac{3}{{\left[Q^{-1}\left(\frac{\epsilon }{8}\right)\right]}^{2}}\cdot SNR\cdot \left(|h_{1}{|}^{2}\right){\left(1+\frac{SNR\cdot \left(|h_{1}{|}^{2}\right)}{\psi_{1}\psi_{2}}\right)}^{a_{N-1}}\right)\\ & =\frac{1}{N}\log\left(\frac{3\cdot SNR\cdot {\left|h_{1}\right|}^{2}}{{\left[Q^{-1}\left(\frac{\epsilon }{8}\right)\right]}^{2}}{\left(1+\frac{SNR\cdot {\left|h_{1}\right|}^{2}}{\psi_{1}\psi_{2}}\right)}^{a_{N-1}}\right), \end{array}$$ (B19)

where $a_{N-1}\approx \left(1-\delta_{F})(N-1\right)$ for sufficiently large *N*. Therefore, the proof is completed.
